# Management Strategies for Dissolved Organic Carbon Reduction from Forested Watersheds using the SWAT-C model

**DOI:** 10.1007/s00267-025-02128-y

**Published:** 2025-02-20

**Authors:** Dongjun Lee, Ritesh Karki, Latif Kalin, Sabahattin Isik, Puneet Srivastava, Xuesong Zhang

**Affiliations:** 1 College of Forestry, Wildlife and Environment, Auburn University, Auburn, AL USA; 2 College of Agriculture and Natural Resources, University of Maryland, College Park, MD USA; 3 USGS Lower Mississippi-Gulf Water Science Center, Tuscaloosa, AL USA; 4 USDA-ARS Hydrology and Remote Sensing Laboratory, Beltsville, MD USA

**Keywords:** Forests, Dissolved Organic Carbon (DOC), Soil and Water Assessment Tool-Carbon (SWAT-C), Management scenarios

## Abstract

Forests serve as crucial carbon sinks, yet quantifying carbon cycle processes within forested watersheds is challenging due to inherent complexities, including multiple carbon pools and variability. Dissolved organic carbon (DOC) transport from forests significantly impacts drinking water quality since it interacts with chlorine to form disinfection byproducts. Although the Soil and Water Assessment Tool-Carbon (SWAT-C) has been widely used to understand carbon fluxes at the watershed scale, the model has been primarily evaluated in non-forested watersheds and loading to aquatic systems, often overlooking terrestrial carbon fluxes from forested regions within watersheds of interests. This study assessed the applicability of SWAT-C in simulating carbon fluxes in both terrestrial and aquatic systems in the forested Big Creek watershed located in the south-central United States (U.S.), which also serves as a drinking water source, and analyzed dominant pathways of DOC transport across the landscape. Additionally, three management scenarios (i.e., forest conversion, raking in forests, and adjusting biomass harvest in croplands) aimed at reducing DOC transport were evaluated. Calibration efforts using remotely sensed as well as datasets demonstrated the proficiency of SWAT-C in simulating both terrestrial and aquatic carbon fluxes in forest-dominated regions. Results emphasize the importance of initializing and calibrating the parameters of dominant land use/cover types to enhance model performance in simulating carbon fluxes. The study found that all evaluated management scenarios can reduce DOC transport into streams, with the conversion of the dominant loblolly pine forests to restored longleaf pine forests achieving a 40% reduction in forest-derived DOC yields. These findings offer valuable insights for watershed-scale carbon cycling modeling and inform management strategies in forest-dominant watersheds to mitigate DOC yields.

## Introduction

Forests, covering 4.06 billion hectares or 31% of the global land surface area, are a vital component of the global terrestrial ecosystem and impart major influence on global biogeochemical cycle. Forests also provide diverse ecosystem services (Martín-López et al., 2016) including enhancements in water quantity and quality (Hassan et al., 2005; Liu et al., 2021), carbon sequestration and storage (Jackson et al., 2005), and climate regulations (Vauhkonen and Packalen, 2018). For instance, forests contribute over 70% of the freshwater supply (Millennium ecosystem assessment, 2005) and store more than 80% of terrestrial aboveground carbon and over 70% of soil organic carbon (SOC) (Batjes, 1996; Dixon et al., 1994). As such, changes in forest structure or composition as well as loss of forest land through natural or anthropogenic causes such as wildfires, increase in harvest frequency and intensity, or changes in forest management practices, can have detrimental impacts. These could include land degradation, water quality impairment due to increase in sediment and carbon transport, and loss of above and belowground carbon stock (Baulenas, 2021; Skulska et al., 2020).

Increasing losses of total organic carbon (TOC) from terrestrial systems into receiving water bodies as well as the atmosphere is a critical ecosystem concern as it not only reduces land productivity and contributes to global warming but also leads to water quality degradation (Köhler et al., 2008; Mattsson et al., 2009). Specifically, the transport of dissolved organic carbon (DOC) from forest ecosystems has profound implications for both climate change and the quality of drinking water resources. Increases in DOC concentration in river systems can indicate substantial changes in terrestrial carbon reserves (Worrall et al., 2004). In aquatic systems, although DOC itself is not considered a pollutant, its excess presence can have adverse effects on surface water quality by facilitating the co-transport of harmful contaminants, such as toxic heavy metals, through complexations (Lawlor and Tipping, 2003). In addition, DOC can contribute to the formation of disinfection byproducts (DBPs) during drinking water quality treatment (Chowdhury et al., 2009; Valdivia-Garcia et al., 2019). With these challenges, understanding fluctuations in DOC concentrations within streams and forested watersheds has become increasingly important (Butman et al., 2016; Karki et al., 2023).

Improved understanding of the sources as well as the fate and transport of DOC at a watershed scale is important for developing science-based decision tools and management strategies aimed at reducing DOC losses from terrestrial landscapes into receiving waters (Du et al., 2019). For this, process-based watershed-scale models can be vital tools as they can help simulate the spatial as well as temporal variability in DOC fluxes in surface waters, facilitating a better understanding of the sources and predictions of changes in DOC fluxes over time. These models can also prove instrumental in comprehending how natural and anthropogenic drivers, such as climate and watershed management, can influence DOC yields from different land use/cover (LULC) types, offering valuable insights for sustainable management. The Soil and Water Assessment Tool (SWAT) is a physically-based, semi-distributed, hydrologic and water quality model that has been widely adopted to simulate hydrologic processes and pollutant loads across various watersheds and environmental conditions (Neitsch et al., 2011) as well as to assess impacts of management strategies (Niraula et al., 2013), LULC alterations (Ahiablame et al., 2019; Lee et al., 2019), and climate change (Kang and Sridhar, 2018), among many scenarios (Karki et al., 2020; Lin et al., 2022). The enhanced version of the model, SWAT-Carbon (SWAT-C) (Zhang et al., 2013), incorporates coupled carbon, nitrogen, and phosphorus cycles derived from three agroecosystem models that include the CENTURY model (Parton et al., 1994), its daily version, DAYCENT (Del Grosso et al., 2001), and the Environmental Policy Integrated Climate (EPIC) model (Izaurralde et al., 2006), allowing SWAT to be utilized for simulating and assessing changes to carbon dynamics including particulate and dissolved form of SOC and fluxes at a watershed-scale. Validation efforts of the SWAT-C model, conducted through simulating CO_2_ fluxes between atmospheric and terrestrial systems at 16 eddy covariance flux towers, provide a robust foundation for simulating terrestrial carbon cycling in soil layers (Zhang et al., 2013). Previous studies employing SWAT-C have provided significant insights into understanding the impacts of climate change and extreme storm events on variations in riverine DOC concentration originating from terrestrial environments (Du et al., 2019; Mukundan et al., 2023). Nonetheless, these studies have predominantly focused on calibrating DOC in streams or vegetation metrics like leaf area index (LAI), overlooking vertical carbon fluxes within terrestrial systems. While some studies have extended to include vertical carbon flux calibration within forest systems (Yang and Zhang, 2016), they were still restricted to the field scale. It is important that carbon levels in streams are influenced by the carbon transported from the surrounding terrestrial environments. Thus, it is imperative to extend the evaluation of SWAT-C in forested watersheds, including the identification of strategies to calibrate spatially variable carbon stock and fluxes in the terrestrial regime as well as lateral carbon fluxes, given that forests constitute a substantial portion of many watersheds. To address this gap, this study assessed the SWAT-C model for its ability to simulate vertical as well as lateral carbon fluxes from forested watersheds. The study also evaluated the use of spatially scaled remote sensing data, including LAI, Net Primary Productivity Carbon (NPPC), and evapotranspiration (ET) as well as measured in-situ carbon data in constraining the vertical as well as lateral fluxes from forested watersheds. The use of these datasets can be vital to capturing the spatial as well as temporal variability in carbon sequestration, storage, and losses by forested systems.

The focus in the evaluation of management strategies for reducing carbon transport from terrestrial landscapes has also predominantly centered on agricultural lands with minimal emphasis on forested landscapes (Drewniak et al., 2015; Lal, 2007; Lugato et al., 2014). DOC dynamics in agricultural landscapes exhibit significant differences compared to those observed in forests (Bhattacharya and Osburn, 2020; Liu et al., 2023). While it is acknowledged that DOC transported from agricultural areas surpasses those from forests (Humbert et al., 2020), it is important to recognize that forests can also be potential primary source areas for DOC in many watersheds due to their extensive coverage. Furthermore, inadequate management practices in forest lands can exacerbate the transfer of DOC from terrestrial to aquatic systems (Jonsson and Wardle, 2010). This study represents the first attempt to quantitatively evaluate forest management strategies specifically aimed at reducing DOC loss from forest-dominant watersheds. Herein, both agricultural and forest management strategies were evaluated.

The overall goal of this study was to evaluate the SWAT-C for its ability to simulate carbon fluxes in a forest-dominated watershed and assess management strategies for reducing DOC losses to receiving water. The specific objectives of this study were to: (1) test SWAT-C for its ability to simulate carbon dynamics in a forest-dominated watershed including the use of remote sensing data products to constrain the spatial and temporal variability in carbon fluxes, (2) understand the dominant pathways of DOC transport, and (3) evaluate management strategies for reducing DOC load to receiving water bodies. Findings from this study contribute to understanding the vertical and lateral carbon fluxes in forest-dominated watersheds as well as expanding comprehension of practical applications of management strategies for reducing contaminant transport related to DOC.

## Materials and Methods

### Study Area

**Fig. 1:**
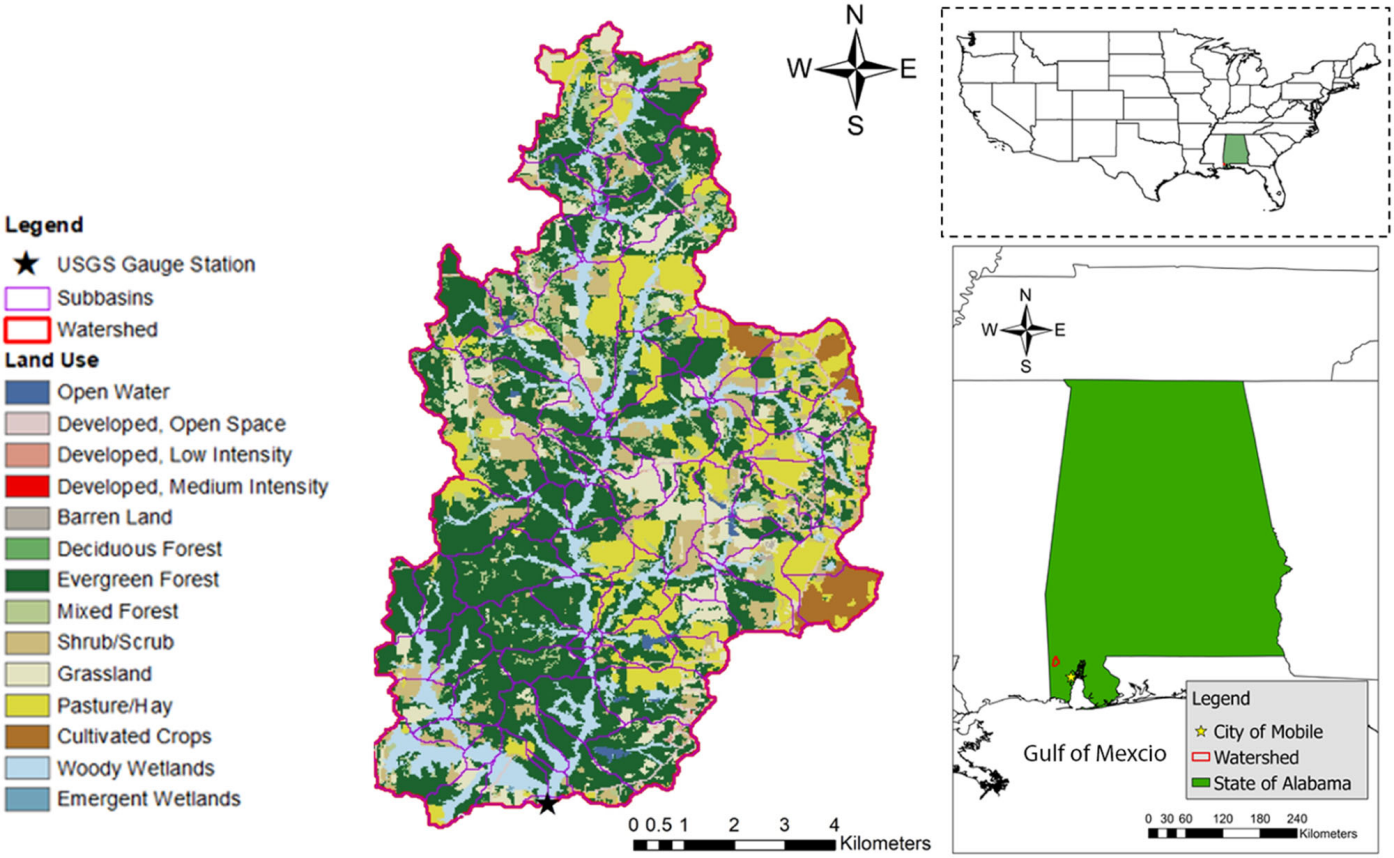
The location of the Big Creek watershed and LULC distribution

The case study was performed in the Big Creek watershed, a headwater watershed located in Alabama, a southeastern state in the United States (U.S.). It is one of the major tributaries to a drinking water reservoir in the City of Mobile in Alabama, contributing 50% of the gauged inflow to the reservoir (Journey et al., 1995). Figure 1 illustrates the location of the Big Creek watershed along with the U.S. Geological Survey (USGS) gauge used for streamflow and TOC calibration and validation. The Big Creek watershed is about 81 km^2^ and is dominated by forests, which cover over 47% of the total area. Following forests, hay and forested wetlands are the other major LULC types accounting for 14% and 12% of the watershed, respectively. It is important to note that forested wetlands identified by LULC maps are mostly located along stream corridors, indicating that the forested wetland consists mostly of riparian areas rather than permanent wetland areas. Based on the Soil Survey Geographic Database (SSURGO), the watershed has two major soil types (i.e., AL213 and AL221) that account for 66% and 32% of the watershed, respectively. The soil AL213 belongs to Hydrologic Soil Group (HSG) D, which is characterized by high runoff potential, while soil AL221 belongs to HSG A, which is characterized by low runoff potential. The watershed receives an annual average precipitation of 1716 mm and the air temperature ranges from −6 to 32 °C.

The reservoir, referred to as the Big Creek Lake, supplies drinking water for the City of Mobile through the Mobile Area Water and Sewer Services. Concerns regarding the water quality of the reservoir as a source of drinking water have promoted numerous scientific investigations (ADEM, 1996; Elias et al., 2014). Specifically, it is essential to assess DOC sources and mitigate their losses from the identified sources due to the potential of DBPs being formed during the drinking water treatment process (Elias et al., 2013). DBPs are formed when organic carbon reacts with chlorine during the disinfection process. The Big Creek watershed was selected as a study area in this study due to its forest-dominated LULC and its role as a drinking water source.

### SWAT-C Model and Model Setup

#### SWAT-C Model

SWAT is a process-based, semi-distributed, watershed-scale model developed to simulate the impact of LULC changes, land management practices, and climate variations on water resources, sediment transport, and agricultural chemical movements within a watershed (Arnold et al., 2012). The model partitions a watershed into multiple subbasins and employs hydrologic response units (HRUs), characterized by areas of homogenous LULC, slope, and soil attributes within each subbasin, as the fundamental modeling units (Neitsch et al., 2011). It simulates the hydrologic process (i.e., runoff, infiltration, lateral flow, groundwater recharge, ET) and pollutant response (i.e., sediment, nutrients, and pesticides) from HRUs to the main channel of each subbasin and routes it down the watershed via the stream network. Details on the governing equations in the SWAT model for simulating hydrology and water quality are provided in the SWAT Theoretical Documentation (Neitsch et al., 2011). The default SWAT model, however, lacks modules for the simulation of terrestrial and aquatic carbon fluxes (Zhang et al., 2013).

To address this limitation, Zhang et al. (2013) developed an enhanced version of the SWAT model by incorporating the CENTURY carbon model to simulate soil organic matter dynamics, resulting in the development of SWAT-C. The current version of SWAT-C (Ver. 683) represents the coupled terrestrial and aquatic carbon cycles at the watershed scale, encompassing terrestrial carbon cycling processes in the soil layers and its transport to streams via diverse pathways, including surface runoff, lateral flow, percolation, and groundwater recharge (Zhang et al., 2013). In addition to the terrestrial processes, in-stream DOC processes such as labile and refractory DOC kinetics and bottom algal processes have been adapted from QUAL2K and CEQUAL-W2 (Zhang et al., 2013). The comprehensive capabilities of SWAT-C, which represent a significant advancement in modeling the interconnected terrestrial and aquatic DOC cycles at the watershed scale, has been assessed in multiple studies (Du et al., 2019; Yang and Zhang, 2016).

An important limitation in the current version of the SWAT-C model when simulating forests includes the disconnect between the model simulated Net Primary Productivity (NPP) and forest biomass assimilation that allowed SWAT-C to count assimilated NPP even without an increase in forest biomass when the simulated biomass had reached the user-defined maximum value. This error could lead to an overestimation of forest NPP and was rectified by adding a check in the source code that allowed for assimilated NPP to be counted only when there was an increase in forest biomass from the previous day. The “grow” subroutine was modified, and additional information regarding the source code modification is provided in Supplementary material (Section 1.1).

#### SWAT-C Model Setup

The primary input into the SWAT model consists of geospatial and meteorological data. The required geospatial dataset includes a digital elevation model (DEM) for topographic information, LULC information, and soil maps with soil physical properties. For this study, a 30 m x 30 m DEM sourced from the National Elevation Dataset (NED) was used as the elevation dataset (U.S. Geological Survey, 2019), a 2019 National Land Cover Database (NLCD) dataset was used for the base LULC information (Dewitz, 2021), and the Soil Survey Geographic Database (SSURGO) provided by the USGS was used for soil properties (NRCS, 2008).

The meteorological data includes air temperature, relative humidity, shortwave radiation, wind speed, and precipitation. The Parameter-elevation Regressions on Independent Slopes Model (PRISM) climate data of daily precipitation and maximum and minimum air temperature on a 4-km grid were used as meteorological input to drive the model (Daly et al., 2008). As PRISM only provides precipitation and temperature dataset, the remaining variables, including daily relative humidity, shortwave radiation, and wind speed were prepared using the North American Land Data Assimilation System-Phase2 (NLDAS-2) climate dataset (Xia et al., 2012). The details of the model input and initialization data used in this study are provided in Table 1. A 10%-10%-0% threshold for LULC, soil, and slope, respectively, was used to define the HRUs which resulted in a SWAT model that consists of 83 subbasins and 290 HRUs for the study watershed. The developed model was run for a period of 32 years from 1990 to 2021 with a warm-up period of two years.

**Table 1:** Datasets used for model development, initialization of major LULC types, and model calibration

	Data	Description	Source
Model Input	DEM	National Elevation Dataset at 30 meters resolution	United States Department of Agriculture (USDA) Geospatial Data Gateway (https://datagateway.nrcs.usda.gov/)
	LULC	2019 NLCD	United States Department of Agriculture (USDA) Geospatial Data Gateway (https://datagateway.nrcs.usda.gov/)
	Soil	Soil Survey Geographic Database (SSURGO)	United States Department of Agriculture (USDA) Web Soil Survey (https://websoilsurvey.sc.egov.usda.gov/)
	meteorological data	Precipitation and Maximum/Minimum air temperature	Northwest Alliance for Computational Science and Engineering (https://prism.oregonstate.edu/)
		Daily humidity, shortwave radiation, wind speed data	Land Data Assimilation System (LDAS) (https://ldas.gsfc.nasa.gov/nldas)
Model Initialization	Biomass	Forest Carbon Stocks and Fluxes, Conterminous USA, 1990–2010	EARTH DATA (https://daac.ornl.gov/cgi-bin/dsviewer.pl?ds_id=1829)
Model Calibration	LAI	A 4-day composite LAI dataset with 500 meters resolution	Google Earth Engine (https://developers.google.com/earth-engine/datasets/catalog/MODIS_061_MCD15A3H)
	NPPC	An 8-day composite NPPC dataset with 500 meters resolution	Google Earth Engine (https://lpdaac.usgs.gov/products/mod17a2hv006/)
	ET	An 8-day composite ET dataset with 500 meters resolution	Google Earth Engine (https://lpdaac.usgs.gov/products/mod16a2v006/)
	Streamflow	Daily discharge from USGS 02479945 gage station	USGS National Water Information System (https://waterdata.usgs.gov/nwis)
	TOC	USGS gage stations	

Forest assimilation of biomass and, therefore, carbon sequestration as well as losses through foliage, is heavily dictated by forest species as well as age (Karki et al., 2023). As the forest was the major LULC in the study watershed and, therefore, imparted major influence on the sediment, nutrient, and carbon dynamics, it was important to accurately initialize the forest age and biomass for the major forest types. This was especially vital to overcome the demonstrated limitations of the SWAT model in accurately simulating forest systems, which is based on algorithms developed for simulating row crops (Haas et al., 2022b). Initial biomass and forest age for the dominant forest types, which included evergreen forests and forested wetlands, were derived from the National Forest Carbon Monitoring System (NFCMS) for forest initialization (Williams et al., 2020). In addition, intra-annual variability in LAI derived from remote sensing data product was utilized to initialize the number and timing of harvest operations for hay LULC in the study watershed. Three harvest operations were applied to the hay, and their parameters (i.e., HARVEFF and HIOVR) were calibrated (Table S1).

### Model Calibration and Validation

The terrestrial hydrologic and carbon dynamics significantly influence carbon cycling in aquatic systems as terrestrial carbon is a major source of riverine carbon (Gentine et al., 2019). It was, therefore, important to make sure that the terrestrial hydrologic and carbon processes were accurately simulated for the major LULC types in the study watershed to constrain the lateral fluxes of carbon from land to streams before constraining the riverine hydrology and carbon processes. For evaluating the terrestrial dynamics in the forested watershed, LAI, ET, and NPPC were selected as calibration components. LAI and ET indirectly represent the biomass and hydrologic processes, while NPPC reflects the vertical carbon dynamics, including carbon storage in biomass and losses to the atmosphere.

Model calibration of the selected variables in the developed model was performed using SWAT-CUP, a calibration and uncertainty analysis tool developed for SWAT (Abbaspour et al., 2007). The Sequential Uncertainty Fitting (SUFI-2) algorithm, which accounts for all uncertainty sources through parameter uncertainty (i.e., a set of parameter ranges), was used for model calibration (Khoi and Thom, 2015; Rouholahnejad et al., 2014) and uncertainty analysis based on p-factor (percentage of observed data that falls within the 95% prediction uncertainty) and r-factor (the average thickness of the uncertainty band) values. A p-factor value close to 1 and an r-factor value close to 0 indicate low uncertainty (Abbaspour et al., 2007). Nash-Sutcliffe efficiency (NSE) was used as the objective function for calibration processes.

Model calibration for LAI and NPPC, the simulation of which are interconnected within the modeling framework, was first performed for the dominant LULC types. These included evergreen forest (FRSE), forested wetlands (WETF), and hay (HAY). This was followed by the calibration for ET simulation. As the observed remote sensing data for LAI, ET, and NPPC were available at a subbasin-scale, we first analyzed the LULC composition for each subbasin to identify subbasins with the highest LULC % for each of the LULC types. The selected subbasins for FRSE, HAY, and WETF cover 100%, 100%, and 61% of the subbasin LULC type, respectively. As a result, the model was first calibrated for FRSE followed by HAY and WETF. As the majority of the remaining LULC in the subbasin selected for WETF was FRSE, the selected sequence for calibrating the major LULC types allowed for better constraining of the model simulation for WETF LULC. The simulated ET, LAI, and NPPC were calibrated using MODerate resolution Imaging Spectroradiometer (MODIS) estimated global ET (MOD16A2GF), LAI (MCD15A3H), and NPPC (MOD17A2H) datasets, respectively (Table 1). All remote sensing datasets used in the study have a spatial resolution of 500 m × 500 m and temporal scales of 4- or 8-days. If a subbasin was covered by multiple grids, the average value was used, whereas, for subbasins covered by a single grid, the value from that grid was directly utilized. Google Earth Engine (GEE) and Python scripts were employed to extract values and aggregate monthly data for model calibration and evaluation. Initial parameters utilized for the calibration of ET, LAI, and NPPC were derived from (Haas et al., 2022a; Haas et al., 2022b).

After calibration of LAI, ET, and NPPC for the three selected subbasins, the calibrated parameters were applied to all corresponding LULC HRUs. The simulated LAI, ET, and NPPC were then assessed at the basin scale for all forest-dominated subbasins as well as the entire watershed to ensure that the model adequately represented both the basin-scale spatial and temporal variability in LAI, ET, and NPPC at the basin scale. Calibration and evaluation of the terrestrial processes were followed by calibration of streamflow and TOC, which was performed using observed datasets from USGS station 02479945, which drained the entire watershed. Due to the absence of observed DOC, observed TOC data was used as a substitute for model calibration. Average observed TOC concentration was 5.05 mg/l, with values ranging from 1.7 to 11 mg/l. Since continuously observed TOC data was not available, the Load Estimator (LOADEST) tool was used to generate monthly TOC loading data from discrete samples and was used for model calibration (Runkel et al., 2004). Reliability of the LOADEST estimated TOC load was ensured by comparing the LOADEST estimated TOC loads with the observed discrete samples (Fig. S1). Details on the parameters calibrated for the terrestrial and riverine processes are provided in Table S1.

As the time periods for which the remote sensing data products and observed streamflow and TOC loadings were available varied, the model was calibrated and validated for the different simulated components for different periods (Table 2). LAI, NPPC, and ET were calibrated and validated utilizing remotely sensed data over a twenty-year period from 2001 to 2020. Streamflow was calibrated and validated using observed streamflow datasets from 1994 to 2020. For TOC loadings, the calibration and validation period spanned only from 1997 to 2004 due to the limited availability of observed data. The availability of remotely sensed datasets began in the early 2000s, while the spatial model input datasets, including LULC, were generated more recently (2019). As such, the model calibration periods for the different variables were selected to align with the timeframe of data availability and generation. Specific details regarding calibration and validation periods for each component are shown in Table 2.

**Table 2:** Model calibration and validation periods for LAI, NPPC, ET, streamflow, and TOC, all of which were performed on a monthly time scale

Components	Simulation period	
	Calibration	Validation
LAI	01-2011 ~ 12-2020	07-2002 ~ 12-2010
NPPC		01-2001 ~ 12-2010
ET		
Streamflow	01-2009 ~ 12-2020	01-1994 ~ 12-2008
TOC	01-2001 ~ 08-2004	11-1996 ~ 12-2000

Model performance evaluation for the simulation of different variables was performed using graphical as well as statistical measures. Statistical measures used for the evaluation included the NSE, the coefficient of determination (R^2^), and percentage bias (PBIAS). NSE, R^2^, and PBIAS are commonly used metrics for the evaluation in hydrologic and water quality modeling studies (Moriasi et al., 2015). NSE ranges from -∞ to 1 with 1 indicating a perfect model fit and values less than 0 indicating that the mean of the observed dataset is a better predictor than the model. R^2^ ranges from 0 to 1 with 0 indicating no correlation between the observed and simulated values while 1 indicating a perfect correlation. PBIAS indicates the average tendency of the simulated value to be larger to smaller than the corresponding observed variable with values closer to 0 indicating accurate model prediction (Moriasi et al., 2015).

### Evaluation of SWAT-C for the Simulation of Carbon Fluxes

As one of the major objectives of this study was to evaluate the capability of SWAT-C to simulate carbon fluxes in forests, the simulated NPPC in forest-dominated subbasins was compared with NPPC estimates derived from remote sensing. The forest-dominated subbasins were identified as subbasins that had more than 60% LULC as forests. NPPC simulations at the basin scale were also compared with the remote sensing estimated NPPC. These allowed for the evaluation of SWAT-C for the simulation of NPPC not only in forest-dominated regions but also for the whole watershed. Annual NPPC values from 2001 to 2020 derived from both SWAT-C and MODIS were compared.

Assessment of NPPC simulation was followed by the evaluation of DOC transport from different hydrologic pathways from forests to inland water bodies. Identification of the dominant pathways for DOC transport from forests to inland water bodies is vital to identifying mitigation strategies for reducing DOC loading from forests. This evaluation was also performed for the remaining major LULC types (WETF and HAY) along with agricultural land (AGRR), although it made less than 3% of the watershed. By comparing DOC transport mechanisms through the different hydrologic pathways, such as surface flow, lateral flow, and groundwater, among these LULC categories, the study aimed to elucidate potential differences in carbon transport dynamics and identify any notable patterns or trends across the landscape.

Total DOC yields and DOC yields per unit area for the major LULC types were also analyzed to identify the LULC types that are most likely to contribute DOC from the landscape to inland water bodies within the study area. Additionally, the influence of the two predominant soil types (AL213 and AL221) on DOC yields was also assessed to determine how the soil types and their properties influenced DOC yields. These analyses can facilitate the development of effective management strategies aimed at reducing DOC at the watershed scale.

### Critical Source Area Analysis and Scenario Development for Reducing DOC Loading

#### Identifying Critical Source Areas (CSAs)

Implementation of management strategies for reducing DOC loading first required the identification of critical source areas (CSAs) for the major LULC type that contribute most of the DOC loadings to the inland water bodies. To identify CSAs contributing to DOC yields within forest regions, FRSE HRUs were ranked based on their DOC load per unit area. By employing this approach, the study aimed to identify key areas within FRSEs that significantly contribute to DOC yields in the study area, facilitating targeted management strategies for mitigating carbon transport. Assessment of CSA was not performed for agricultural lands as it covered only 3% of the watershed. Forests and agricultural lands were selected as the LULC types for the development and assessment of management scenarios as these have been identified as the major LULC types for DOC export to inland water bodies (Kaushal et al., 2014; Bhattacharya and Osburn, 2020; Liu et al., 2021).

#### Scenario Development

The SWAT model has been widely implemented for assessing the effectiveness of management practices at the watershed scale (Neitsch et al., 2011). However, previous studies have primarily focused on evaluating agricultural management practices (Oeurng et al., 2011; Tian et al., 2023), with limited attention given to forests. This study evaluated the effectiveness of three management scenarios in reducing DOC yields for both forests and agricultural lands. The details of the management strategies evaluated in this study are provided in Table 3.

**Table 3:** The agricultural and forest land scenarios in reducing DOC

Scenarios	Target HRUs	Description	Methodology
Baseline	–	The calibrated model	
Forests conversion Scenarios	FRSE HRUs exhibiting the highest 20% yields of DOC	Convert evergreen forests to range-brush	Convert the parameters related to land use and plants
		Convert evergreen forests to range-grasses	
		Convert evergreen forests to long-leaf trees	
Forests Raking scenario		Raking residue of forests	Change the model source code to increase the removed forest residue from 10% to 90% with 10% interval
Agricultural Scenarios	AGRR HRUs	Increase the aboveground biomass that is removed when harvesting	Increase the fraction of aboveground biomass removed in harvest operations (HVSTI) by 30%
			Increase the HVSTI by 50%
			Increase the HVSTI by 70%

The first scenario was a LULC change scenario that involved converting FRSE to other land use types that had the potential for reducing DOC loading when compared to loadings from forests. The study transformed the identified CSAs within FRSEs into two distinct rangeland types (i.e., Range-Brush (RNGB) and Range-Grasses (RNGE)) and restored longleaf pine by adjusting parameters related to LULC (*.hru and *.mgt) and plant growth *(plant.dat*). Restored longleaf pine is a native pine species of southeastern U.S. that has less biomass density than traditional southern pines and is more resistant to climate change and fire. The calibrated parameters for simulating restored longleaf pine were acquired from Haas et al. (2024).

The second scenario involving FRSE LULC considered raking of litterfall to reduce biomass lost from trees to the land surface through litterfall at the onset of dormancy and ranged from 10% to 90% of total litterfall from the critical source forest areas. This approach required modification of the SWAT-C source code to implement the raking scenario which was implemented by reducing the fraction of biomass (raked biomass) that is added to the soil layer based on the % removed at the onset of dormancy. For example, if the raking ratio is 10%, 90% of the foliage will be added to the soil layer while 10% will be removed. The “dormant” subroutine was modified, and additional information about the modification of the SWAT-C source code is provided within Supplementary material (Section 1.2).

Agricultural lands are recognized as significant sources of DOC due to the presence of crop residues (Mailapalli et al., 2013). Accordingly, an additional scenario was evaluated focused on agricultural LULC which involved increasing removal of crop residues left within agricultural lands at harvest. Increasing removal of crop residue during or after harvest operations can potentially help decrease DOC yields by reducing the amount of biomass available for decomposition. The agricultural scenario increased residue harvested by 30%, 50%, and 70%, which was simulated by adjusting the HVSTI parameter that defines the fraction of aboveground biomass removed in harvest operations.

Assessment of the changes in DOC yields from forest and agricultural LULC was performed by comparing baseline DOC loads at the HRU level (field scale) as well as the watershed outlet. Additionally, a t-test was also performed to evaluate the significance of differences in the DOC loadings between the baseline and the evaluated scenarios at the watershed outlet.

## Results

### Model Calibration and Validation

**Fig. 2:**
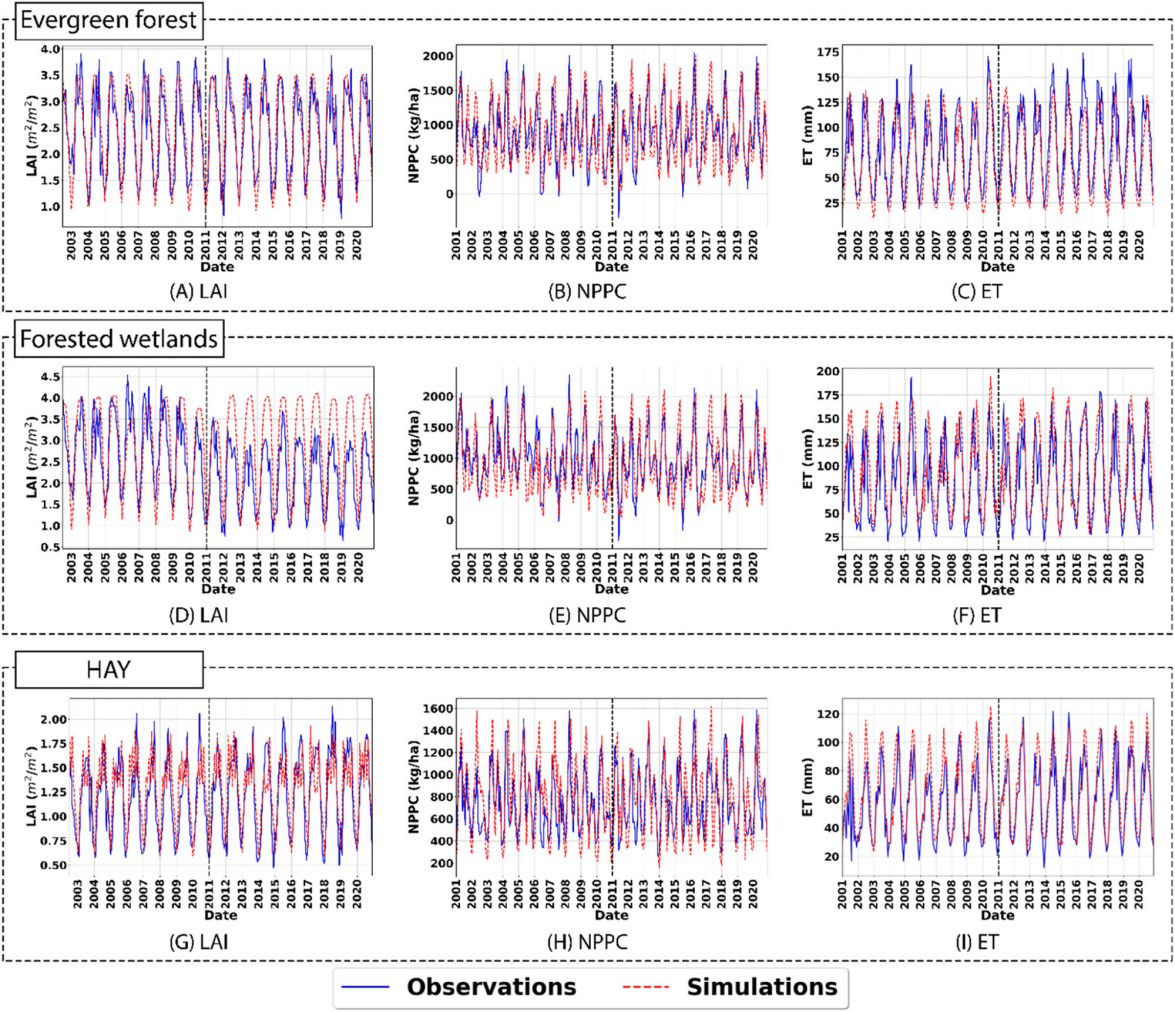
Graphical comparison between monthly simulated and remote sensing estimated **A** LAI, **B** NPPC, and **C** ET for evergreen forests (top row); **D** LAI, **E** NPPC, and **F** ET for forested wetlands (middle row); and **G** LAI, **H** NPPC, and **I** ET for hay (bottom row). The vertical black dashed line distinguishes the calibration and validation period for each LULC type and simulated variable

Model performance evaluation for the simulation of LAI, NPPC, and ET for subbasins dominated by FRSE, WETF, and HAY after calibration and validation showed that SWAT-C performed well in replicating the observed trend and variability for all LULC types except for WETF in the simulation of LAI and ET during the calibration period (Table 4; Figs. 2; S2).

**Table 4:** Model performance for LAI, NPPC, and ET simulations for the subbasin with dominant LULC types and monthly streamflow and TOC loading at the watershed outlet

Calibration level	Components	Model performance					
		Calibration			Validation		
		NSE	R^2^	PBIAS (%)	NSE	R^2^	PBIAS (%)
Evergreen forest dominant subbasin	LAI	0.75	0.78	−2.3	0.74	0.76	−3.3
	NPPC	0.49	0.62	4.0	0.48	0.58	0.8
	ET	0.66	0.78	16.7	0.59	0.68	9.8
Forested wetland dominant subbasin	LAI	−0.47	0.75	−38.2	0.54	0.64	−4.6
	NPPC	0.51	0.69	−0.8	0.47	0.69	15.5
	ET	0.60	0.69	−12.1	0.32	0.54	−18.8
Hay dominant subbasin	LAI	0.61	0.61	−2.3	0.45	0.57	−10.1
	NPPC	0.38	0.55	−2.4	0.28	0.57	−8.0
	ET	0.47	0.68	−16.2	0.75	0.80	−7.3
Watershed outlet	Streamflow	0.68	0.78	3.1	0.52	0.90	−19
	TOC	0.63	0.70	−16.9	0.85	0.87	11.1

Evaluation of LAI, ET, and NPPC simulations of FRSEs showed that SWAT-C was able to simulate all three variables well with R^2^ and NSE > 0.48 and PBIAS less than 20% during both calibration and validation periods. Comparison across the simulated variables showed that the model slightly underperformed in the simulation of NPPC with NSE > 0.48, but R^2^ > 0.58 and PBIAS less than 5% indicated that the model was able to adequately capture the variability and had minimal bias in the simulation of NPPC. Graphical analysis revealed that the SWAT-C model replicated the observed trends for NPPC within FRSE HRUs effectively (Fig. 2B), though it failed to accurately simulate peak values for LAI and ET in some years (Fig. 2A, C). This discrepancy may be attributed to the use of constant parameter values for LAI (e.g., maximum and minimum potential LAI values) throughout the simulation period, which can often be dynamic and can lead to the model failing to accurately capture the observed temporal variability.

The model followed similar trends in the simulation of LAI, ET, and NPPC for HAY LULC. R^2^ and NSE > 0.45 and PBIAS less than 20% showed that the model was able to simulate LAI and ET well. The model, however, had difficulty in the simulation of NPPC with NSE of 0.38 and 0.28 during calibration and validation, respectively, similar to that observed for FRSE LULC. Graphical comparison between the simulated and remote sensing estimated NPPC for HAY showed that the model overestimated NPPC and ET during the summer growing months in some years (Fig. 2H), which led to reductions in the performance metric. Additionally, while the model was able to replicate the overall LAI trend, including both growing season and non-growing seasons over the years, it continued to struggle with capturing peak LAI values (Fig. 2G).

Assessment of the remote sensing LAI data product for the subbasin dominated by WETF showed two distinct trends of LAI with peak LAI close to 4.0 m^2^/ m^2^ before 2011 and closer to 3.0 m^2^/ m^2^ after 2011 (Fig. 2D). The model had difficulty in replicating the two distinct trends in the LAI of WETF resulting in poor model performance for LAI simulations with NSE of −0.47 and PBIAS of −38.2% during the calibration period. As the simulation of ET is highly correlated to LAI simulation in the SWAT-C model, NSE for ET was also poor during the calibration period with a value of 0.32. The model, however, performed well for ET and LAI simulation during the validation period with R^2^ and NSE > 0.5 and PBIAS less than 20%. It was also surprising to see that the model was able to replicate the observed trend and variability for NPPC for WETF for both calibration and validation periods well with NSE > 0.47, R^2^ > 0.65, and PBIAS less than 20% even with the difficulty of SWAT-C in replicating the remote sensing estimated LAI during the calibration period.

After evaluation for specific LULC types, the model simulated LAI, ET, and NPPC was evaluated for the whole watershed (Figs. S3 and S4, Table 5), which showed that the model adequately replicated the spatial as well as temporal variability in the simulation of LAI and ET with NSE > 0.7, R^2^ > 0.8, and PBIAS less than 20% for both calibration and validation periods (Table 5). Similar to that observed for specific land use types, SWAT-C had difficulty replicating the spatial and temporal variability in NPPC with NSE of only 0.21 and 0.16 during the calibration and validation periods, respectively. Graphical evaluation of the basin average simulation of NPPC showed that the model failed to match the observed NPPC trends during the summer months (Fig. S3). But R^2^ > 0.48 and PBIAS less than 27% still showed that the model was able to capture the variability observed in remote sensing estimated NPPC and had minimal bias even when evaluated for the whole watershed. At larger spatial scales, the averaging effect reduces local-scale variability, resulting in better alignment between model predictions and observed data (Gebeyehu et al., 2023). This demonstrates that spatial aggregation can enhance model performance by mitigating local discrepancies and emphasizing broader hydrological trends. Overall, the model performance for LAI, ET, and NPPC at both subbasins and basin scales were acceptable, aligning well with findings from previous studies that evaluate these components within forested landscapes using SWAT (Haas et al., 2022a; Haas et al., 2022b; Karki et al., 2023; Karki et al., 2025).

**Table 5:** Model performance evaluation for the simulation of LAI, NPPC, and ET at the basin scale

Components	Simulation period	Model performance		
		NSE	R^2^	PBIAS (%)
LAI	01-2011 ~ 12-2020	0.81	0.83	1.1
	07-2002 ~ 12-2010	0.77	0.81	1.3
NPPC	01-2011 ~ 12-2020	0.16	0.48	26.6
	01-2001 ~ 12-2010	0.21	0.49	24.7
ET	01-2011 ~ 12-2020	0.78	0.84	12.0
	01-2001 ~ 12-2010	0.76	0.91	17.5

**Fig. 3:**
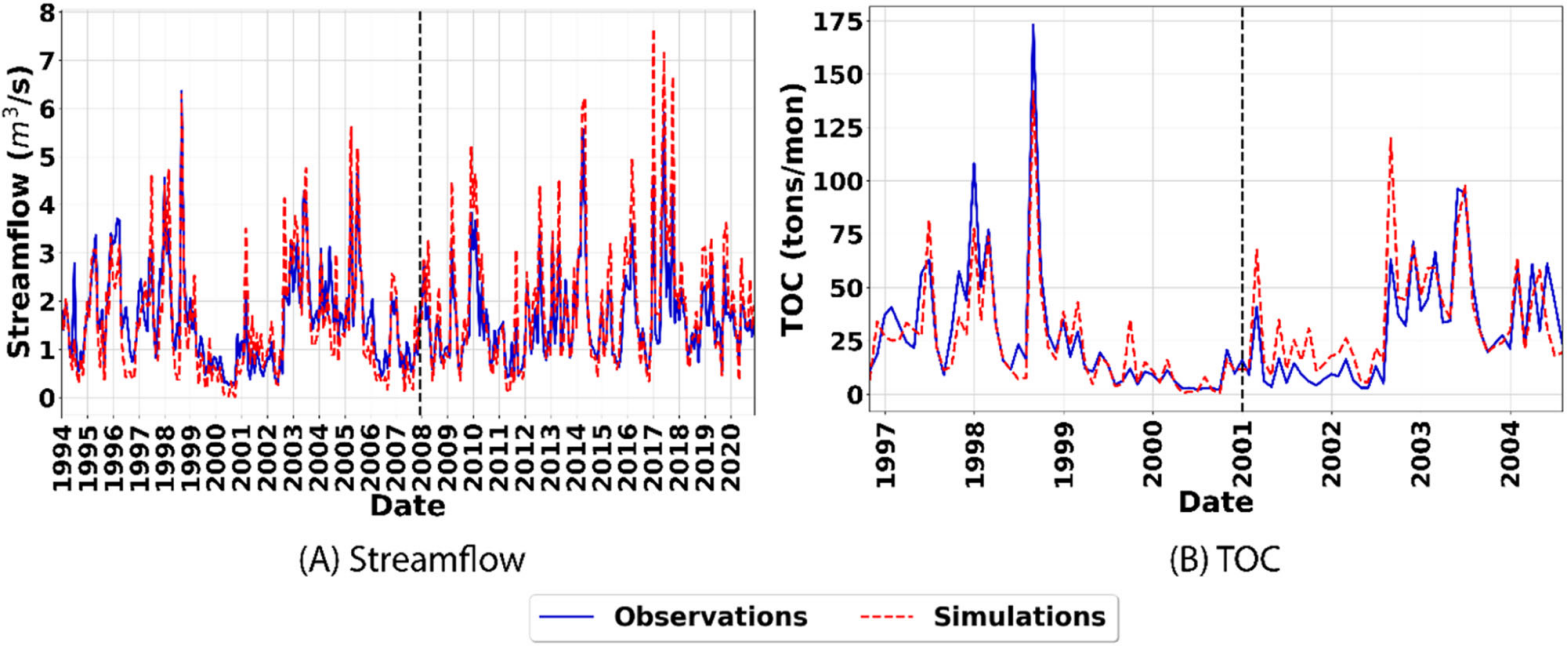
Comparison between **A** simulated and observed streamflow and **B** simulated and observed TOC loading for the study watershed after calibration and validation. The vertical black dashed lines indicate the separation of the periods used for calibration and validation

Model calibration and evaluation of the riverine processes for streamflow and TOC after constraining of the terrestrial processes showed that SWAT-C adequately simulated the observed streamflow and TOC for the study watershed with R^2^ > 0.7, NSE > 0.5 and PBIAS less than 20% during both calibration and validation period (Table 4, Fig. 3) based on metric ranges defined by Moriasi et al. (2015). Graphical evaluation of streamflow and TOC demonstrated that SWAT-C performed well during both low flow as well as high flow periods for streamflow and replicated the temporal variability in observed TOC well. However, the model overestimated streamflow and TOC during high flow periods in the calibration phase (Fig. 3). These overestimations may be attributed to the use of the curve number (CN) approach for flow estimation, which does not explicitly account for rainfall intensity (Garen and Moore, 2005; Grimaldi et al., 2013) and can result in inflated flow as well as TOC predictions.

Assessment of the uncertainty in the simulation of the terrestrial as well as riverine processes by evaluating the p-factor and r-factor values (Table S2) showed that the model had high confidence in the simulation of streamflow and TOC at the watershed outlet (p-factor > 0.73; r-factor < 0.88). The model, however, had higher uncertainty in the simulation of LAI and NPPC for forests and forested wetlands (p-factor < 0.6).

### Evaluation of SWAT-C simulation of carbon fluxes

**Fig. 4:**
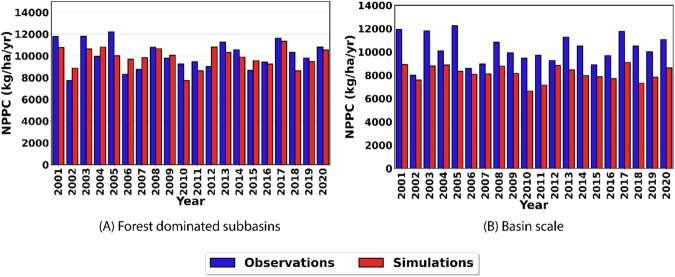
Comparison between observed and simulated annual NPPC values for **A** forest-dominated subbasins, and **B** basin scale

Assessment of SWAT-C simulation of terrestrial carbon fluxes for forests was first performed by comparing the remote sensing estimated vs simulated annual NPPC in forest-dominated subbasins within the study watershed which was followed by evaluation for the whole basin (Fig. 4). Average annual remote sensing estimated NPPC values for the forest dominated subbasins ranged from 7726 kg/ha/yr to 12,197 kg/ha/yr, while the simulated NPPC values ranged from 7741 to 11,350 kg/yr. This indicated that SWAT-C closely replicated NPPC in forest-dominated subbasins with the simulated annual average NPPC of 9874 kg/ha/yr compared to the remote sensing estimated NPPC of 10,065 kg/ha/yr over the whole simulation period. Graphical evaluation showed that SWAT-C was able to replicate the observed trends for NPPC well in years where the NPPC was low but failed to match the productivity in some years with high NPPC (Fig. 4A).

Evaluation of model simulated annual NPPC against the remote sensing estimated at the basin scale showed that the model underestimated NPPC with simulated annual average NPPC of 6,040 kg/ha/yr compared to the estimated NPPC of 10,220 kg/ha/yr over the whole simulation period (Fig. 4B). It is important to note that other minor LULC types within the study watershed were not calibrated for the simulation of LAI, ET, and NPPC. As the evaluation was performed at the subbasin scale and the NPPC simulation in other LULC types contributes to the subbasin average simulated NPPC, this could have contributed to the underestimation of NPPC at the basin scale. It is important to note that the evaluation of subbasins dominated by the major forest types (FRSE and WETF) performed well in replicating the remote sensing estimated NPPC. Despite these limitations, graphical evaluation has shown that the model was able to replicate the temporal variability in the simulation of NPPC well for the study watershed with the simulated NPPC closely following the trend of remote sensing estimated NPPC despite failing to match the magnitude in years with high observed NPPC (Fig. 4).

**Fig. 5:**
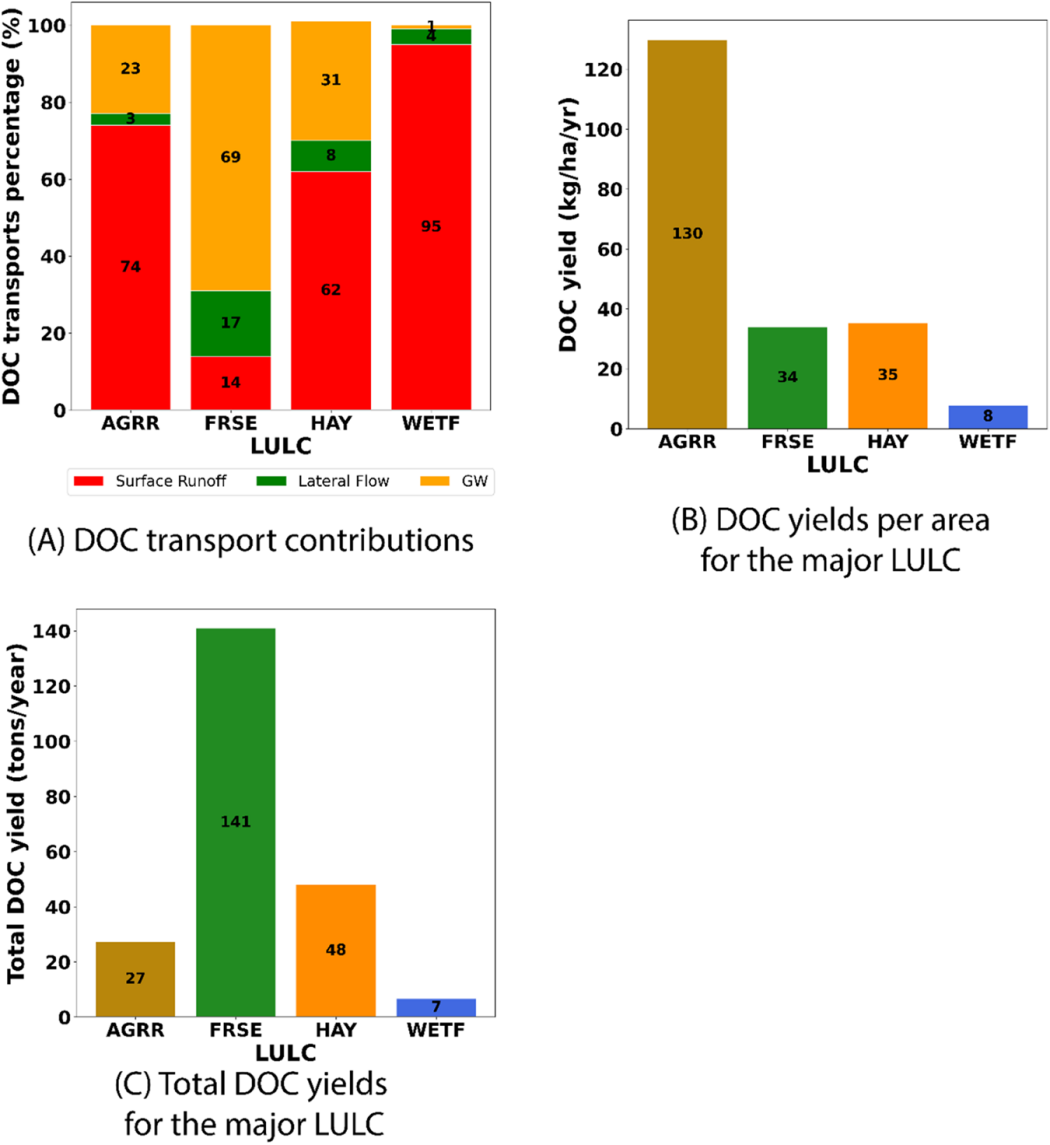
The DOC transport proportions from hydrologic components **A**, DOC yield per area **B**, and the total DOC yield **C** for the major LULC types (AGRR; FRSE; HAY; and WETF)

Assessment of the capability of the SWAT-C model to replicate observed trends in carbon sequestration in forest-dominated watersheds was followed by the identification of the major hydrologic pathways through which carbon was lost from the forest-dominated watersheds to inland waters. Evaluation of the DOC load lost through different hydrologic pathways showed that groundwater flow was the most dominant mechanism through which DOC was lost in FRSEs (69%) while surface runoff and lateral runoff contributed to 17% and 14% of the DOC loads to inland waters from FRSEs, respectively (Fig. 5A). These results, in conjunction with Fig. S5, are consistent with the idea that forest streamflow generally originated from subsurface or groundwater recharge (Fan et al., 2014; Adane and Gates, 2015). Surface runoff, however, was the dominant pathway for DOC transport from WETFs, which accounted for 95% of the DOC load (Fig. 5A). As DOC transport is highly correlated to hydrologic pathways, it helps explain the high DOC transport through groundwater in FRSEs as subsurface flow is the dominant hydrologic pathway in forests due to high infiltration and percolation (Eimers et al., 2008). Similarly, the dominance of surface flow in wetlands dominated by marshes and floodplains explains the high DOC transport through the medium in WETFs. The dominant transport mechanisms were also evaluated for HAY and AGRR as these LULC types can be important sources of DOC to inland waters. It was observed that surface runoff was the dominant pathway for DOC transport in both HAY and agricultural LULC types accounting for 62% and 74% of the total DOC load, respectively. Lateral flow was the second dominant pathway contributing 31% and 23% of the DOC from HAY and agricultural LULC types, respectively. Notably, the DOC transport proportion in agricultural LULC observed in this study is consistent with findings from previous studies that utilized data analysis in predominantly agricultural watersheds (Eckard et al., 2017). As shown in Fig. 3, TOC loads follow the streamflow patterns from 1997 to 2004. Except for FRSE, other LULCs exhibited dominant DOC transport through surface runoff (Fig. 5A), consistent with the observations that the DOC concentrations generally increase with an increase in streamflow (Raymond et al., 2016; Hosen et al., 2021).

Evaluation of DOC yield from the different LULC types showed that agricultural LULC had the highest DOC yield per hectare at 130 kg/ha/yr followed by FRSEs and HAY at close to 35 kg/ha/yr while WETFs had the lowest DOC yield at 8 kg/ha/yr (Fig. 5B). However, estimation of the total DOC yield from the different LULC types showed that FRSE contributes disproportionately to the total DOC yield from the watershed at 141 tons/yr, which was more than 3 times higher than estimated from agricultural lands at 27 tons/yr (Fig. 5C). This is because FRSE is the dominant LULC in the watershed while agricultural lands covered only a small portion of the watershed. HAY was the second dominant LULC type for DOC yield at 48 tons/yr while WETF contributed the least to the DOC yield in the watershed (Fig. 5C). The lower contribution of DOC yield from WETF differs from the previous studies that identify wetland as major source of DOC to rivers and streams (Wei et al., 2021; Zhang et al., 2023). This difference may be attributed to the nature of the forested wetlands in the study watershed, which often function as riparian zones near stream corridors rather than as permanent wetland areas. DOC load accounted for, on average, 55% of the total TOC loads from the different LULC types but ranged from 39% to 86%. This was consistent with findings from similar studies (Sobczak and Rosińska, 2020).

The influence of the two dominant soil types (i.e., AL213 and AL221) on DOC yields for the FRSEs, which exhibited the highest DOC yields in the study area, were also analyzed. The amount of DOC yield from forests with AL221 at 2,382 kg/ha/yr was approximately five times higher than that from forests with AL213 at 478 kg/ha/yr. As higher DOC transport occurs in groundwater in forests (Fig. 4B), the results suggest that AL221, which corresponds to HSG A with a high infiltration rate, has the potential to yield higher DOC than AL213, which is in HSG D.

### Critical Source Area Analysis and Scenario Evaluation

#### Identification of Critical Sources Areas (CSA)

Assessment of the CSAs showed that targeting 50% of the DOC yield originating from the FRSE LULC involved selecting approximately 20% of areas within FRSE HRUs (Fig. S6). Targeting 20% of the forest area as a threshold resulted in the selection of 29 HRUs (8.62 km^2^; 10.6% of the watershed area) which were identified as CSA within forested regions of the watershed. These CSAs were characterized by soil AL221, classified as HSG A, indicating high infiltration rates. This characteristic facilitates substantial DOC transport in groundwater in forested areas (Fig. 5A), resulting in high DOC yields. For agricultural lands, all agricultural HRUs were chosen, as these areas represented only 3% of the total watershed area.

#### Scenarios Impact on DOC Yield at the Watershed-scale

**Fig. 6:**
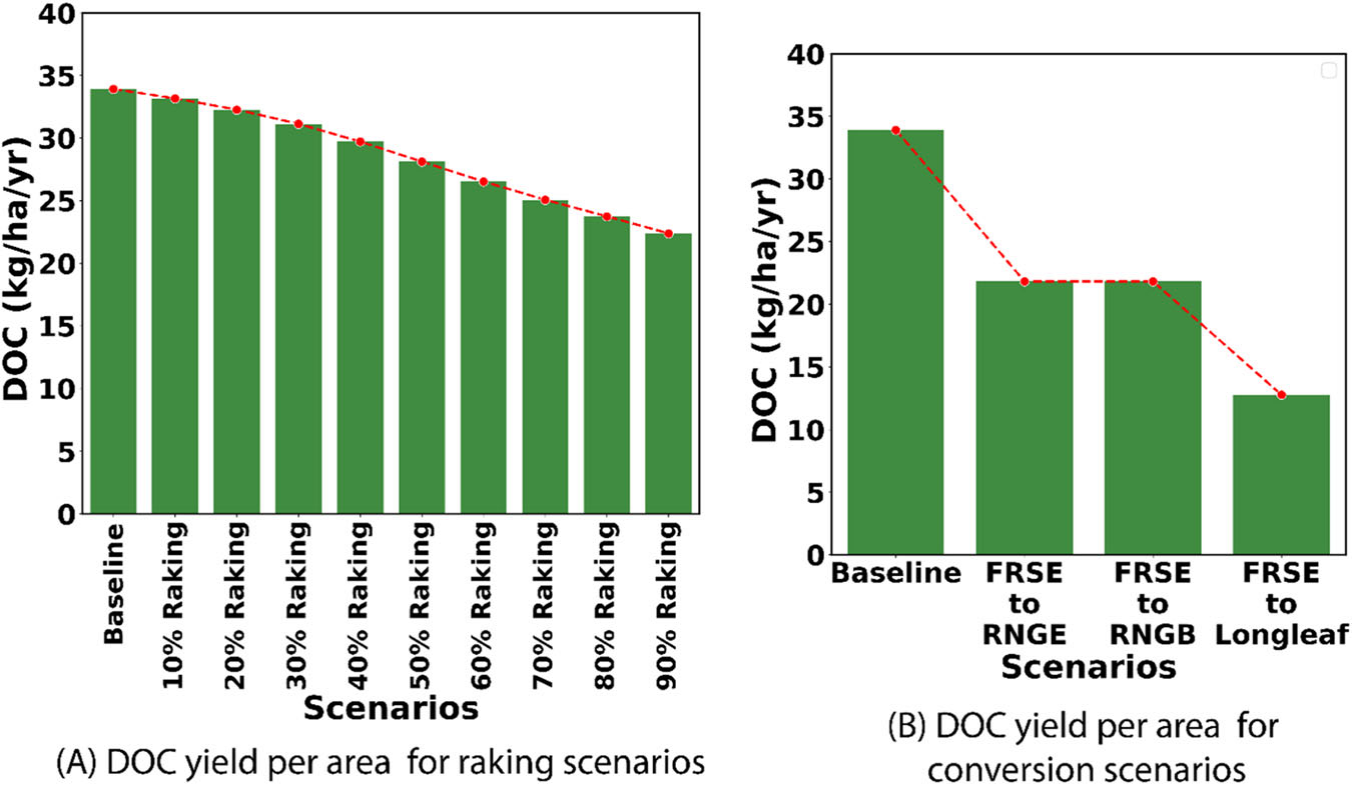
Reduction in DOC load from evergreen forest due to **A** raking of litterfall (left), and **B** conversion to RNGE, RNGB, and restored long-leaf pine LULC types (right)

Estimation of DOC load from CSAs within FRSEs showed an average loss of 33.9 kg/ha/yr. Raking of 10% to 90% of the foliage loss from forest floors towards the end of the growing season showed a linear trend in the reduction of DOC loads from 33.1 kg/ha/yr to 22.3 kg/ha/yr, a reduction of 2% to 34% (Fig. 6A). Although the removal of foliage from forest floors could expose the soil, there was no observed significant increase in sediment loss with the removal of foliage from forests, which could likely be attributed to the dominance of subsurface hydrologic pathways in the CSAs within forest LULC (Fig. S5). Conversion of FRSEs to restored longleaf pine, a forest native to the southeastern U.S., would lead to the biggest reduction in DOC load of 62% from 33.9 kg/ha/yr to 12.7 kg/ha/yr (Fig. 6B). Conversion of FRSEs to RNGB or RNGE (range grasses/brush) in the CSAs would also lead to a considerable reduction of 36% in DOC load to inland waters (Fig. 6B).

**Fig. 7:**
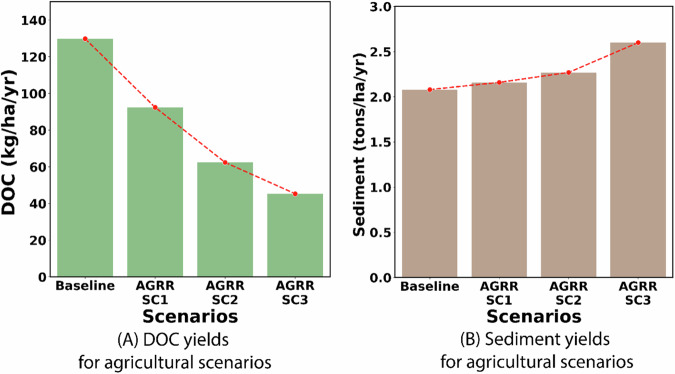
**A** Reduction in DOC loading from agricultural lands due to increasing harvest of crop residue and, **B** Increase in sediment load compared to baseline due to increasing harvest of crop residue. The AGRR SC1, SC2, and SC3 indicate the scenarios that increase harvest residues by 30%, 50%, and 70%, respectively

DOC load from AGRR at 130 kg/ha/yr was almost three times more than estimated for forests at 34 kg/ha/yr, most of which comes from crop harvest residue at the end of the growing season. A reduction in crop harvest residue by increasing the harvested crop biomass by 30%, 50%, and 70% would lead to a DOC load reduction from 130 kg/ha/yr to 92.6, 62.4, and 45.3 kg/ha/yr, respectively, a load reduction of 29%, 52%, and 65% (Fig. 7A). It is, however, important to note that increasing harvest biomass from row crops and thereby, reducing crop residue, would expose the bare soil to natural elements including precipitation and the dominance of surface runoff as the major hydrological pathways in agricultural LULC led to an increase in sediment loss to 2.2, 2.3, and 2.6 tons/ha/yr (for 30%, 50%, and 70% increase in harvest), compared to a baseline loss of 2.1 tons/ha/yr (Fig. 7B).

**Fig. 8:**
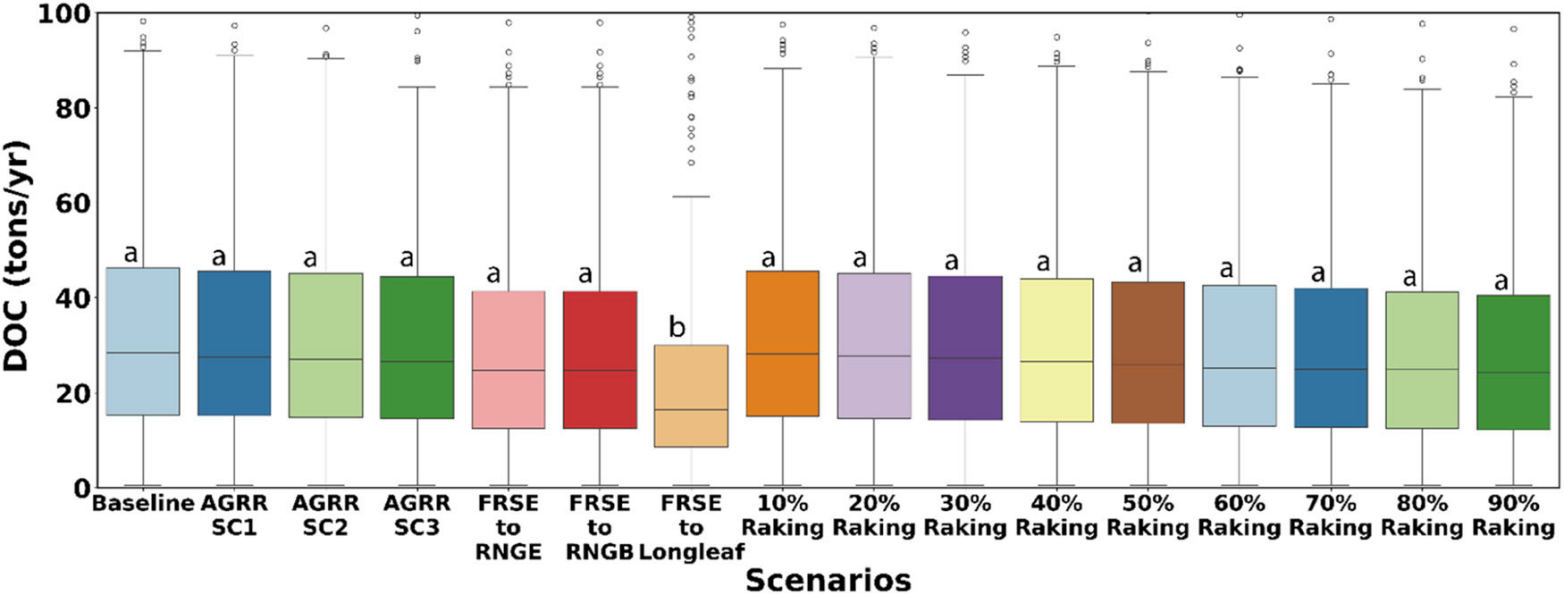
DOC loadings at the watershed outlet for the baseline model and all scenarios. The same letters indicate no significant differences between the two variables while different letters indicate significant differences between the two variables

The changes to DOC load due to all the proposed scenarios were also evaluated at the watershed outlet (Fig. 8). Comparison across all the scenarios showed that conversion from FRSE to restored longleaf pine would lead to the biggest reduction in DOC load of 40% at the watershed outlet from 39.3 tons/yr to 23.6 tons/yr. It was also the only scenario that showed a significant difference in DOC load at the watershed outlet when compared to the baseline (*p* < 0.05). Reductions in DOC load due to conversions of FRSEs to rangelands (RNGE/RNGB) were close to 8% while the raking of foliage would result in DOC load reduction from 1–10% but was not statistically significant (*p*-value ranged from 0.16 to 0.93). Similarly, the increase in biomass harvest from agricultural lands by 30%, 50%, and 70% would reduce DOC load by 2%, 4%, and 5% at the watershed outlet, respectively, but showed no significant difference when compared to the baseline (*p*-values from 0.27 to 0.77).

## Discussion

### Carbon Flux Simulations in SWAT-C

Assessment of the SWAT-C at the landscape level showed that the model can adequately replicate the magnitude as well as temporal variability in the simulation of NPPC in forests. It was, however, important to note that SWAT-C could potentially overestimate assimilated NPP in forests when the forest reaches the user-defined maximum biomass value and needed adjustment to the source code that allowed for NPP assimilation only when there was an increase in forest biomass. The model also performed well in replicating the NPPC for HAY LULC but it was important to alter the management practices for the timing and number of harvest operations using signals of change in LAI from remote sensing datasets to simulate the observed trends in NPPC as well as LAI and ET. This also explains the slight difficulty SWAT-C had in replicating the remote sensing estimated NPPC for HAY compared to FRSEs as it is difficult to represent the annual variability of these practices in the model.

Assessment of SWAT-C for the simulation of WETFs highlighted the difficulty the model can have in replicating two distinct temporal trends in terrestrial processes in natural LULC types (like WETFs) that can happen due to sudden changes in land characteristics that are not captured by the model. The SWAT model uses a base LULC map and often utilizes incorporated management operations to capture the temporal variability in terrestrial processes that can influence LAI, ET, and NPPC, which can be difficult to attain for natural landscapes. This issue can be compounded by the fact that SWAT allows for only a single plant type to be simulated in each HRU. As a result, the model can have difficulty replicating observed changes in LAI, ET, and NPPC due to forest succession or the combined influences of multiple plant types in mixed LULC types such as WETFs. This was highlighted by the difficulty of SWAT in simulating LAI and ET for WETFs. The difficulty of SWAT to accurately simulate carbon fluxes in mixed LULC types was also reported by Karki et al. (2023). The influence of the model’s inability to simulate NPPC in mixed LULC types was also evident in the basin scale evaluation of NPPC as the model underestimated NPPC at the basin scale while performing well when evaluated for individual LULC types.

The ability of SWAT-C to simulate NPPC reasonably well even with poor model performance for the simulation of LAI for WETFs highlights the deficiency of SWAT-C in linking the simulation of LAI with NPPC. In SWAT, plant productivity is based on accumulated heat units and the influence of LAI in plant productivity can only be captured indirectly through the simulation of ET and plant water stress. However, inaccurate and, especially, under-representation of LAI can lead to a simulation of low plant water stress due to low simulated PET, and, therefore, the model can fail to capture its influence in NPPC simulation.

This study also highlights the importance of utilizing remote sensing data products in accurately simulating the spatial as well as temporal variability in terrestrial processes. Many studies have integrated remote sensing LAI, ET, and NPPC data products for constraining the hydrological processes in SWAT (Haas et al., 2022a; Haas et al., 2022b; Karki et al., 2023). Given the complexity of the carbon processes, the chemical loading, including TOC, from terrestrial landscapes can vary due to small differences in geology, soil, streambed materials, or prevailing climate patterns (Hasenmueller et al., 2015). As such, the use of remote sensing data products to constrain the spatial variability in the simulation of terrestrial processes is vital to identifying CSAs for carbon losses for targeted implementation of mitigation measures. Accurate representation of terrestrial processes can also help improve the simulation of riverine hydrology and carbon losses as evidenced by good model performance for the simulation of streamflow and TOC at the watershed outlet, which is vital to accurately estimate carbon loads from the watershed outlet under baseline as well as proposed mitigation scenarios. Additionally, parameter sensitivity results identified erosion of POC (er_POC) and percolation coefficient of DOC (Peroc_DOC) as the two most sensitive parameters for the simulation of TOC (Table S3). This can provide valuable insights for future studies involving the simulation of TOC or identifying methods to reduce TOC losses from forested watersheds.

Assessment of the dominant pathways for DOC transport showed that groundwater flow was the most dominant mechanism for DOC loss from FRSEs (Fig. S3), while surface runoff was the dominant mechanism for WETFs. Surface runoff was also the dominant mechanism for DOC transport for HAY and agricultural LULC types. These results, which are consistent with similar studies in agricultural areas (Dalzell et al., 2011; Du et al., 2023), demonstrate the model’s capability to accurately replicate the dominant transport mechanisms for DOC across agricultural and forest landscapes. As shown in Fig. 3, TOC loads follow the streamflow patterns from 1997 to 2004, consistent with the observations that the DOC concentrations generally increase with rising streamflow (Raymond et al., 2016; Hosen et al., 2021). It is also important to note that surface flow may significantly contribute to peak TOC loads in the stream. Evaluation of the DOC load from the different LULC types showed that agricultural LULC had the highest DOC loss rate of close to 130 kg/ha/yr, almost three times higher than for FRSEs and HAY. This resulted in agricultural lands contributing 27 tons/yr of DOC at the watershed outlet, which is 12% of the total load while only covering 3% of the watershed area. However, forests contributed the highest DOC load at the watershed outlet at 63% due to their larger coverage in the study watershed. And, as it was evident from the CSA analysis that 20% of FRSEs contribute disproportionately to the DOC load, it is important to develop and assess management scenarios with a focus on CSAs on forested watersheds for reducing DOC loading to the watershed along with evaluation of management measures for agricultural LULC.

### Scenario Assessment in Reducing DOC Load

Previous studies on DOC transport have predominantly focused on agricultural watersheds, given their role in mobilizing soil-derived dissolved organic matter and DOC into streams (Kaushal et al., 2014). Similarly, studies assessing management practices using SWAT have primarily focused on agricultural practices, such as controlling residues, vegetation filter strips, and soil conservation. For example, Lupwayi et al. (2006) demonstrated that conventional tillage management leads to faster wheat and peanut residue decomposition compared to no-till practices. However, DOC export from agricultural land does not always exceed that from natural or unmanaged systems (Don and Schulze, 2008; Werner et al., 2021). This variability highlights the need for careful consideration of crop and soil types when evaluating DOC transport dynamics. Likewise, forest management, including harvesting, reforestation, thinning, racking, and conversions, have been proposed in previous studies to improve water quality (Arthur et al., 1998; Pike et al., 2010), but their effectiveness in reducing DOC has not been adequately quantified. This study fills this gap by providing a quantitative assessment of the reduction of DOC load from forest-dominated watersheds through forest management practices as well as conversion by performing a case study in the Big Creek watershed of the southeastern U.S.

Accurate predictions of hydrologic responses using models are needed to establish a plan for efficient watershed management, as inaccuracies can lead to physical and economic losses (Yang et al., 2020). Thus, this study employed the SWAT-C model that was calibrated for the terrestrial processes of LAI, ET, and NPPC simulation for the dominant LULC types as well as streamflow and riverine TOC at the watershed outlet to quantitatively evaluate management practices aimed at reducing DOC yield and loading. Findings from the evaluation of scenarios aimed at FRSEs showed that both raking of forest foliage as well as conversion to restored longleaf pine forests or rangelands can help reduce DOC export. However, these forest management strategies have significant impacts on nutrient cycles and ecosystem services, leading to the degradation of soil and water quality and wildlife habitat (Mulia et al., 2021; Kooch et al., 2021). Notably, converting FRSEs to restored longleaf pine is the most effective method for reducing DOC export in the southeastern U.S. As restored longleaf pine has less biomass density than FRSEs in the region, the reduced loss of carbon from foliage describes the reduction in DOC export. It is also important to note that components that cannot be simulated by the SWAT-C model including improved resistance to pests and better adaptability to climate extremes including droughts which can help reduce tree mortality in restored longleaf pines can also contribute to reducing DOC export from forests in the southeastern U.S. (Qi et al., 2022). The economic consequences of converting FRSEs that are predominantly loblolly pine to longleaf pine (Susaeta and Gong, 2019), however, could be a hindrance to the implementation of this scenario. Reducing harvest residue by increasing harvested biomass in agricultural lands also showed the potential to reduce DOC export and thereby contribute to reducing DOC loading at the watershed outlet. However, adoption of these scenarios could lead to other unfavorable outcomes including an increase in sediment loss due to increased exposure of soil as well as potential loss of soil productivity due to soil quality degradation due to reduction in soil organic matter. It is, therefore, important to note that the scenarios were developed and evaluated to reduce DOC in the study watershed as it serves as a drinking water source. Also, when considering these scenarios as management practices, the characteristics of the watersheds and the purpose of the management scenarios, as well as the potential disadvantages, such as soil degradation and increased sediment loss, should be considered.

### Study Limitations

Although one of the objectives of this study was to assess methods to reduce DOC loading from forested watersheds, this study utilized TOC loads for constraining the simulation of riverine carbon processes instead of DOC due to the lack of available DOC data for the watershed. Similarly, the availability of TOC data as discrete samples for a relatively short time period necessitated the utilization of LOADEST, a widely used tool for generating continuous water quality loads (Spencer et al., 2009; Oh and Sankarasubramanian, 2011), to estimate continuous TOC loads. Although LOADEST estimated values were used for model calibration, comparison between observed grab samples and LOADEST estimated TOC loads showed strong agreement (Fig. S1), allowing for high confidence in the model simulated results. Additionally, successful calibration and validation of terrestrial carbon fluxes over a twenty-year period was also vital to help reduce uncertainty in predicting carbon fluxes in both terrestrial and aquatic environments.

The SUFI-2 algorithms used in the model calibration provided some insight into the uncertainty of the model results. However, SUFI-2 treats all sources of uncertainty uniformly, it can be difficult to identify the source of uncertainty when using diverse data types and a multivariable calibration approach. In contrast, data assimilation (DA) can explicitly handle uncertainties across different observation data types (Salamon and Feyen, 2009), such as remote sensing and in-situ measurements (Bayat et al., 2022; Zafarmomen et al., 2024) and provide better understanding on the source and estimates of uncertainty. Bayat et al. (2023) suggests that DA often outperforms SUFI-2, especially in multivariate calibrations with varying data accuracies. Integrating DA into SWAT-C for assimilating variables like those used in this study could enhance model performance and robustness by effectively addressing uncertainty.

Another limitation of our study is that SWAT-C is primarily designed for assessing management practices in agricultural areas, lacking an internal module for evaluating forest management strategies. To address this limitation, we modified the SWAT-C source code to accommodate forest management strategies. However, the absence of dedicated forest management modules underscores the need for further development. Future research should focus on developing specialized modules within SWAT-C to effectively assess a wide range of management strategies (e.g., selective logging, thinning, extending harvest rotations, and construction of riparian buffer zones, etc.) commonly employed in forest lands. This will enhance the model’s applicability and accuracy in simulating forested watershed dynamics and management practices.

Implementation of these management strategies assessed in this study poses another challenge due to the inherent difficulty of managing natural forests, which demands significant labor or costs (Keenan, 2015). For instance, while raking in commercial forests may be relatively straightforward, it becomes considerably more challenging in natural forests. Additionally, this study focused solely on the reduction of DOC load in streams. Further assessments are required when implementing management strategies, including evaluating impacts on water and nutrient yields and other ecosystem services such as wildlife habitats. The removal of crop residues could also necessitate additional machinery or labor. To effectively implement these management strategies, incentive and assistance programs are essential to support farmers in coping with the added costs and logistical demands.

## Conclusion

This study aimed to assess the applicability of the SWAT-C model to simulate carbon fluxes in terrestrial and aquatic systems in a forested watershed and understand the dominant pathways of DOC transport across various LULC types in the watershed. Moreover, management strategies for reducing DOC loading across forest and agricultural LULC types were also quantitatively evaluated.

Findings from the study indicate that SWAT-C demonstrates proficiency in simulating terrestrial carbon fluxes and TOC loading in forested watersheds. Accurate carbon cycle modeling in forested watersheds necessitates proper initializations of dominant forest types (i.e., FRSE and WETF) as well as the use of remotely sensed data products including LAI, NPPC, and ET to constrain the terrestrial processes for the major LULC types in the study watershed. While the model effectively replicated remote sensing estimated NPPC values in forest-dominant subbasins, it underestimated the NPPC values at the basin scale. This discrepancy is attributed to the challenges of accurately simulating NPPC across multiple plant types within each HRU (Karki et al., 2023) and the potential presence of LULC types that were not calibrated for the simulation of NPPC. Despite these challenges, calibrating NPPC simulations for the dominant LULC types with remotely sensed data helped reduce uncertainty in carbon flux predictions within the forested watershed. Additionally, the discrepancies in DOC transport between agricultural, forests, and wetlands underscore the model’s capability to replicate carbon flux dynamics in diverse landscapes, allowing the identification of CSAs and targeted management strategies for mitigating DOC transport.

The study also evaluated three management strategies aimed at reducing DOC transport in both forests and agricultural LULC types. Despite the absence of internal modules for evaluating the management strategies, we modified the SWAT-C source code to incorporate forest management strategies. Our findings revealed that all scenarios resulted in a reduction in DOC yield from inland areas into streams. Notably, the conversion to restored longleaf pine forests exhibited a significant decrease in DOC loading, leading to a 40% reduction in DOC yield from forests. Statistical analysis indicated that there were no significant differences in DOC transport and loading among the other scenarios.

The modeling approaches combined with remote sensing data used in this study enable accurate simulations of carbon cycle processes in forested watersheds. The identification of dominant pathways of DOC transport across various LULC types provides valuable insights into potential differences in carbon transport dynamics and helps understand notable patterns or trends across the landscape. Moreover, the evaluation of the management strategies aimed at reducing DOC loadings can offer valuable assistance to landowners and forest managers in comprehending effective forest management strategies in forested watersheds, which play a crucial role as important drinking water resources.

## Supplementary information

Supplementary information

## Data Availability

No datasets were generated or analysed during the current study.
