# Supplementary material for: Management Strategies for Dissolved Organic Carbon Reduction from Forested Watersheds using the SWAT-C model
Source: Environ Manage. 2025 Feb 20;75(5):1181–200. doi: 10.1007/s00267-025-02128-y (PMC12033212; doi:10.1007/s00267-025-02128-y)
Supplement: Supplementary file 1 — Supplementary information [file 267_2025_2128_MOESM1_ESM.docx]

Evaluation of Management Strategies for Dissolved Organic Carbon Reduction in a Forested Watershed using the SWAT-C model

**1. SWAT-C Fortran source code (Rev. 683) modifications**

- 1. Overview and modification of the grow (grow_change.f90) subroutine

The “grow” subroutine adjusts plant biomass, leaf area index, canopy height, and NPPC while accounting for the effect of water, temperature, and nutrient stresses on the plant. The model categorizes plants into seven categories: warm season annual legume, cold season annual legume, perennial legume, warm season annual, cold season annual, perennial, and trees. Based on the plant types, the subroutine estimates biomass, leaf area index, canopy height, and NPPC accordingly.

The modified code allows for assimilated NPP to be counted only when there was an increase in forest biomass from the previous day. A new variable, “bio_ms_last_day”, was added to track the biomass of the previous day. The modified code is shown below.

bio_ms_last_day: the variable keeping track of biomass of the last day

bio_ms_last_day = bio_ms(j)

if(idc(idp) == 7) then

if (bio_ms(j) > bio_ms_last_day) then

NPPC_d(j) = NPPC_d(j) + bioday * reg* CFB

else

NPPC_d(j) = NPPC_d(j)

end if

else

NPPC_d(j) = NPPC_d(j) + bioday * reg* CFB

end if

- 1. Overview and modification of the dormant (dormant_change.f90) and allocated_parms(allocate_parms_change.f90) subroutines

The “allocate_parms” subroutine allocates array sizes for parameters in the SWAT-C model. The “dormant” subroutine checks the dormant status of different plant types. The “bio_leaf(ikplt(ihru))” parameter indicates the fraction of biomass that drops during dormancy for trees only. This parameter is defined at the LULC level, meaning that all HRUs with the same LULC share identical values. This prevents adjustments for specific HRUs. To address this limitation, we modified the parameter definition from the LULC to HRU levels, changing it to “bio_leaf(ihru).” We then adjusted the parameter values for HRUs identified through CSA analysis. When the raking scenarios were applied to the model, the bio_leaf was reduced. For example, if the raking ratio is 10%, 90% of the foliage will be added to the soil layer while 10% will be removed.

-------------------------------------------------------------------------------------------------------

In the “allocated_parms” subroutine

mhru: number of HRUs

bio_leaf(mhru)

-------------------------------------------------------------------------------------------------------

In the “dormant” subroutine

Target_HRU: the list of target HRU that was identified by CSA analysis

Ratio of raking: ratio of raking (0.1 to 0.9)

case (7)

idorm(j) = 1

resnew = 0.

target_HRU = (list of target HRU number)

if (j == target_HRU) then

bio_leaf(j) = bio_leaf(j) * (1-ratio of raking)

endif

resnew = bio_ms(j) * bio_leaf(j)

-------------------------------------------------------------------------------------------------------

**Table S1.** Calibrated parameters and descriptions for LAI, NPPC, ET, streamflow, and TOC

| Component | Parameter | | Description | Units | Ranges | Evergreen Forests | Wetlands | Hay | Basin-scale |
| --- | --- | --- | --- | --- | --- | --- | --- | --- | --- |
| LAI & NPPC | V_LAI_INIT | | Initial leaf area index |  | 0.1~ 0.5 | 0.5 | 3.5 | 0.1 |  |
|  | V_BIO_INT | | Initial dry weight biomass | kg/ha | 260 ~ 56,000 | 47,000 | 56,000 | 260 |  |
|  | V_PHU_PLT | | Total number of heat units or growing degree days needed to bring plant to maturity. |  | 3,000 ~ 12,000 | 5000 | 7658 | 3471 |  |
|  | V_BIO_E | | Radiation-use efficiency or biomass-energy ratio | (kg/ha)/  (MJ/m2) | 13 ~ 20 | 17.88 | 15.35 | 15.73 |  |
|  | V_BLAI | | Maximum potential leaf area index | m^2^/m^2^ | 2~ 4.5 | 3.53 | 4.13 | 2.22 |  |
|  | V_FRGRW1 | | Fraction of the plant growing season or fraction of total potential heat units corresponding to the 1^st^ point on the optimal leaf area development curve |  | 0 ~ 0.15 | 0.11 | 0.01 | 0.09 |  |
|  | V_DLAI | | Fraction of growing season when leaf area begins to decline | m^2^/m^2^ | 0.15 ~ 1 | 0.29 | 0.59 | - |  |
|  | V_PLTNFR | | Nitrogen uptake parameter #1-#3: normal fraction of nitrogen in plant biomass at emergence, 50% maturity, and maturity |  | 0.0001 ~0.03 | - | - | 0.01~0.03 |  |
|  | V_T_OPT | | Optimal temperature for plant growth | °C | 10 ~ 25 | 16.12 | 16.73 | 18.03 |  |
|  | V_T_BASE | | Minimum (base) temperature for plant growth | °C | 1 ~ 5 | 2.50 | 3.71 | 0.86 |  |
|  | V_ALAI_MIN | | Minimum leaf area index for plant during dormant period | m^2^/m^2^ | 0.6 ~ 0.8 | 0.86 | 0.74 | 0.58 |  |
|  | V_BIO_LEAF | | Fraction of tree biomass accumulated each year that is converted to residue during dormancy |  | 0 ~ 1.0 | 0.11 | 0.18 | 0.45 |  |
|  | V_MAT_YR | | Number of years required for tree species to reach full development | years | 100 ~ 500 | 259 | 140 | - | - |
|  | V_BMX_TREES | | Maximum biomass for a forest | tons/ha | 700 ~ 1,200 | 290 | 742 | - | - |
|  | V_BMDIEOFF | | Biomass die-off fraction |  | 0~ 0.5 | 0.19 | 0.38 | - | - |
|  | V_HARVEFF | | Harvest efficiency |  | 0.90 ~ 0.95 | - | - | 0.95 | - |
|  | V_HI_OVR | | Harvest index override | (kg/ha)/ (kg/ha) | 0~ 1.0 | - | - | 0.53 ~ 0.80 | - |
| ET | V_CANMX | | Maximum canopy storage | mmH_2_O | 0 ~ 100 | 4.85 | 7.57 | - | - |
|  | V_ESCO | | Soil evaporation compensation factor |  | 0 ~ 1 | 0.98 | 0.42 | - | - |
|  | V_EPCO | | Plant uptake compensation factor |  | 0 ~ 1 | 0.75 | 0.73 | - | - |
|  | V_GSI | | Maximum stomatal conductance at high solar radiation and low vapor pressure deficit | m/s | 0 ~ 5 | 0.003 | 0.312 | 0.001 | - |
|  | V_VPDFR | | Vapor pressure deficit corresponding to the second point on the stomatal conductance curve |  | 1.5 ~ 6 | 5.96 | 4.357 | 5.84 | - |
|  | V_EXT_COEF | | Light extinction coefficient |  | 0~ 1.99 | 0.63 | 1.94 | 0.69 | - |
|  | V_RDMX | | Maximum root depth | m | 0 ~ 10 | 1.52 | - | - | - |
| Streamflow | R_CN2 | | Initial SCS runoff curve number for moisture condition II |  | -0.05 ~ 0.05 |  |  |  | -0.011 |
|  | R_OV_N | | Manning’s “n” value for overland flow |  | -0.05 ~ 0.12 |  |  |  | 0.112 |
|  | V_SURLAG | | Surface runoff lag coefficient |  | 0.05 ~ 24 |  |  |  | 0.534 |
|  | V_GW_DELAY | | Groundwater delay time | days | 0 ~ 500 |  |  |  | 88.220 |
|  | V_GWQMN | | Threshold depth of water in the shallow aquifer required for return flow to occur | mmH_2_O | 80 ~ 150 |  |  |  | 106.355 |
|  | V_ALPHA_BF | | Baseflow alpha factor | 1/days | 0.02-0.035 |  |  |  | 0.032 |
|  | V_REVAPMN | | Threshold depth of water in the shallow aquifer for “revap” or percolation to the deep aquifer to occur | mmH_2_O | 50~ 150 |  |  |  | 67.850 |
|  | V_GW_REVAP | | Groundwater “revap” coefficient |  | 0.002~0.2 |  |  |  | 0.161 |
|  | V_RCHRG_DP | | Deep aquifer percolation fraction |  | 0.001~ 0.03 |  |  |  | 0.024 |
|  | V_CH_N2 | | Manning’s “n” value for the main channel |  | 0.01-0.1 |  |  |  | 0.077 |
|  | V_CH_K2 | | Effective hydraulic conductivity in main channel alluvium | mm/hr | 0.025~6 |  |  |  | 0.523 |
|  | R_SOL_Z | | Depth from soil surface to bottom of layer | mm | -0.15 ~ 0.15 |  |  |  | 0.044 |
|  | R_SOL_AWC | | Available water capacity of the soil layer | mmH_2_O/ mm soil | -0.15 ~ 0.15 |  |  |  | 0.011 |
|  | R_SOL_K | | Saturated hydraulic conductivity [mm/hr] |  | -0.15 ~ 0.15 |  |  |  | -0.039 |
| TOC | V_SPCON | | Linear parameter for calculating the maximum amount of sediment that can be re-entrained during channel sediment routing |  | 0.0001 ~ 0.01 |  |  |  | 0.005 |
|  | V_PRF_BSN | | Peak rate adjustment factor for sediment routing in the main channel |  | 0 ~2 |  |  |  | 0.715 |
|  | V_SPEXP | | Exponent parameter for calculating sediment re-entrained in channel sediment routing |  | 1 ~ 1.5 |  |  |  | 1.352 |
|  | V_ADJ_PKR | | Peak rate adjustment factor for sediment routing in the subbasin |  | 0.5 ~ 2 |  |  |  | 1.151 |
|  | V_Peroc_DOC | | DOC percolation coefficient |  | 0 ~ 1 |  |  |  | 2.162 |
|  | V_Part_DOC | | Organic carbon partition coefficient |  | 500 ~ 2,000 |  |  |  | 0.349 |
|  | V_hlife_doc | | DOC half-life in groundwater | days | 0 ~ 100 |  |  |  | 911 |
|  | V_er_POC | | POC enrichment ratio |  | 0 ~ 5 |  |  |  | 92.583 |
| Parameter columns: | | R_parameter: multiplies the existing values with (1+the given value) V_parameter: replaces the existing values with the given value | | | | | | | |

**Table S2.** Results of uncertainty analysis for the major LULC and basin-scale components (LAI, NPPC, ET, streamflow, and TOC).

| Calibration-scale | Variables | p-factor | r-factor |
| --- | --- | --- | --- |
| Evergreen forests | LAI | 0.54 | 0.24 |
|  | NPPC | 0.35 | 0.58 |
|  | ET | 0.75 | 0.75 |
| Forested wetlands | LAI | 0.49 | 0.88 |
|  | NPPC | 0.51 | 0.90 |
|  | ET | 0.67 | 0.82 |
| Hay | LAI | 0.81 | 1.65 |
|  | NPPC | 0.52 | 1.37 |
|  | ET | 0.73 | 0.80 |
| Basin-scale | Streamflow | 0.90 | 0.87 |
|  | TOC | 0.73 | 0.87 |
| p-factor: the percentage of observations covered by the 95PPU; a p-factor value close to 1 indicates low uncertainty.  r-factor: the thickness of the 95PPU envelop; an r-factor value close to 0 indicates low uncertainty. | | | |

**Table S3.** Results of sensitivity analysis for TOC parameters

| Component | Parameter | t-Stat | P-value | Sensitivity rank |
| --- | --- | --- | --- | --- |
| TOC | er_POC | -10.236 | 0.000 | 1 |
|  | Peroc_DOC | -3.042 | 0.002 | 2 |
|  | PRF_BSN | 1.152 | 0.250 | 3 |
|  | Part_DOC | -0.586 | 0.558 | 4 |
|  | SPEXP | 0.297 | 0.766 | 5 |
|  | hlife_doc | 0.239 | 0.811 | 6 |
|  | SPCON | 0.173 | 0.863 | 7 |
|  | ADJ_PKR | 0.000 | 0.999 | 8 |
| t-Stat: a measure of the relative sensitivity of a model parameter. A higher absolute value of the t-Stat indicates that the parameters is more influential or sensitive in affecting the model output  P-value: the probability that the observed sensitivity of a parameter. A smaller p-value indicates that the parameter is statistically significant. | | | | |


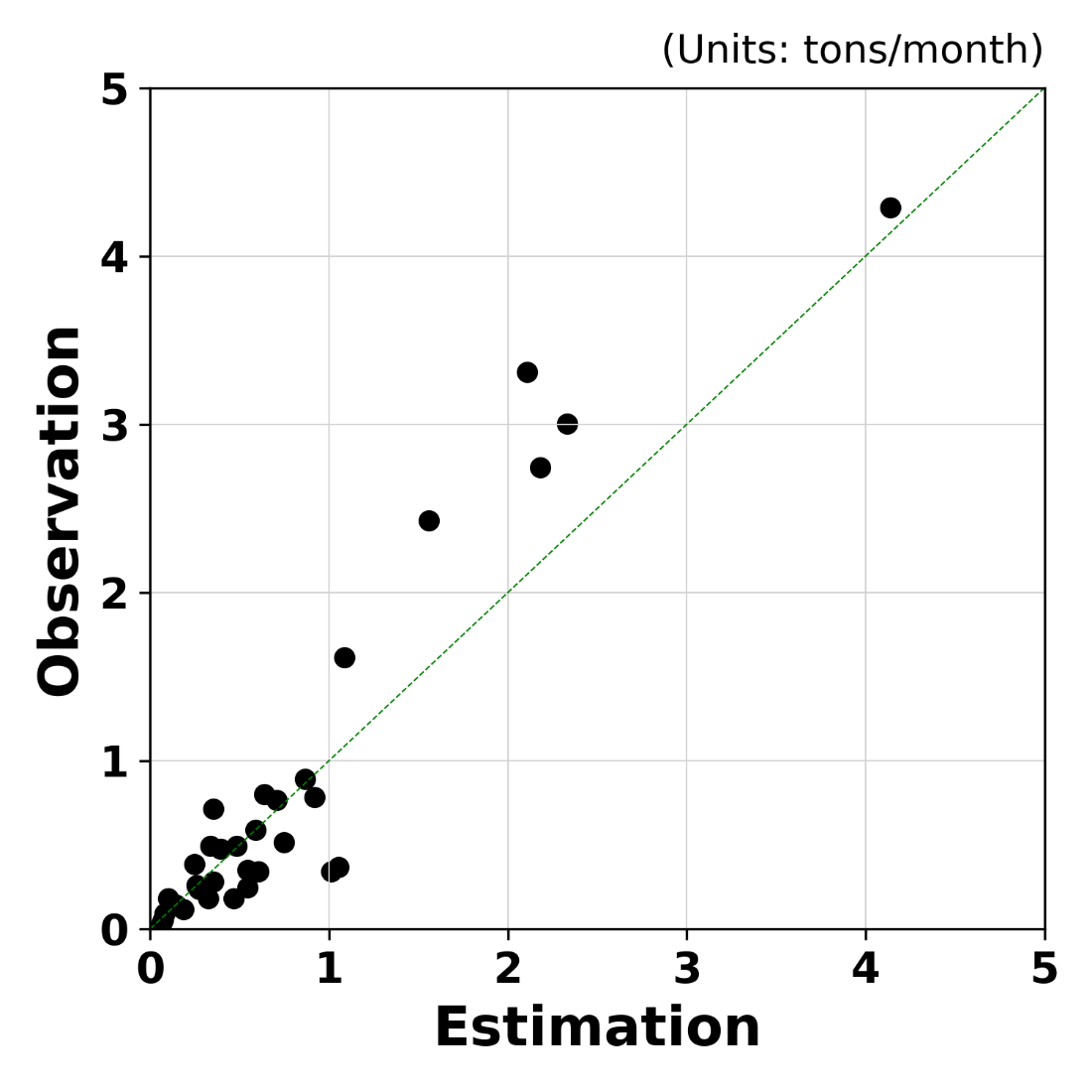


**Figure S1.** The scatter plot comparing observed and estimated TOC loads using LOADEST, illustrating the agreement between observations and model predictions.


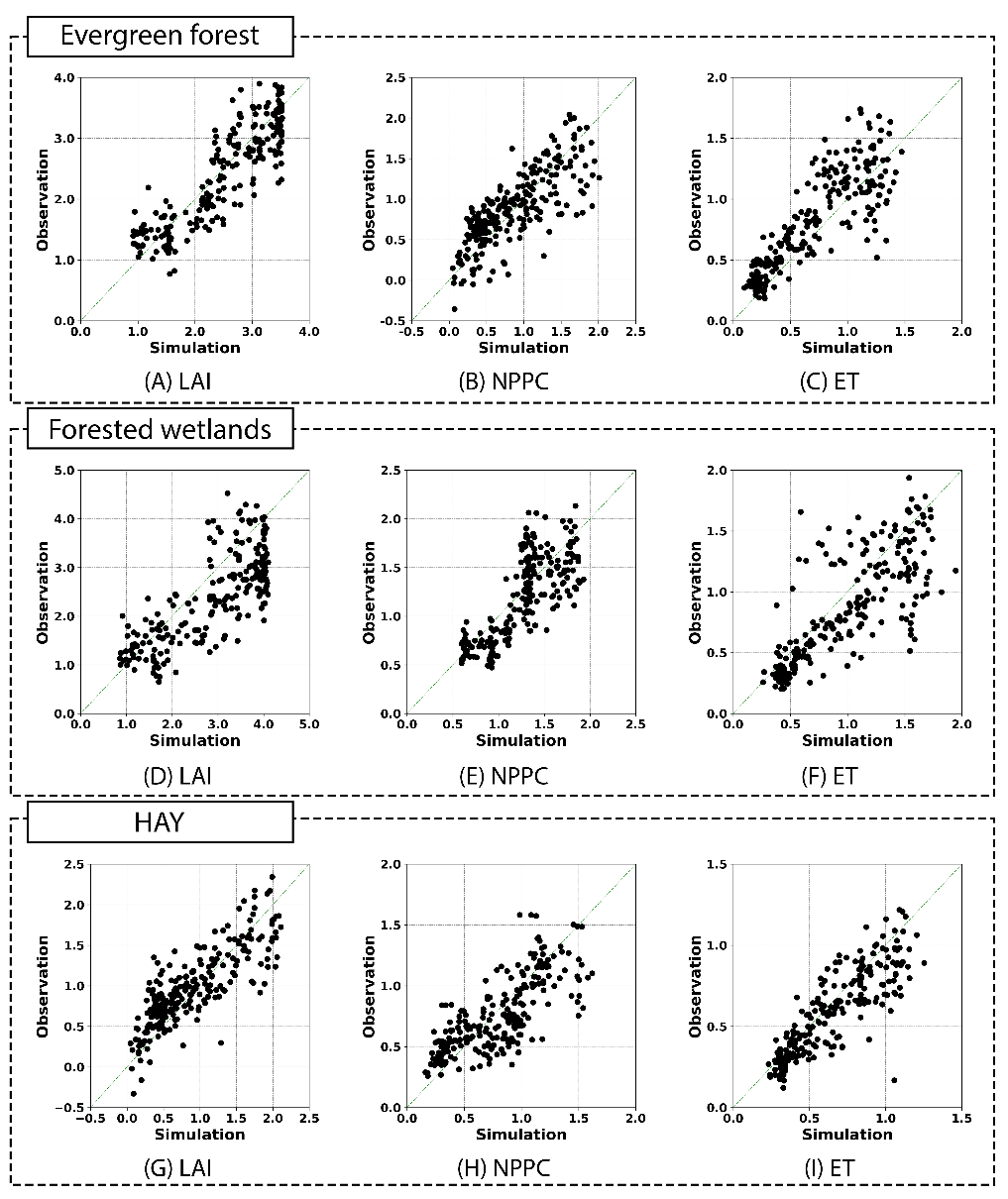


**Figure S2.** Scatter plots between monthly simulated and remote sensing estimated LAI, NPPC, and ET for evergreen forests (top row), forested wetlands (middle row), and hay (bottom row).


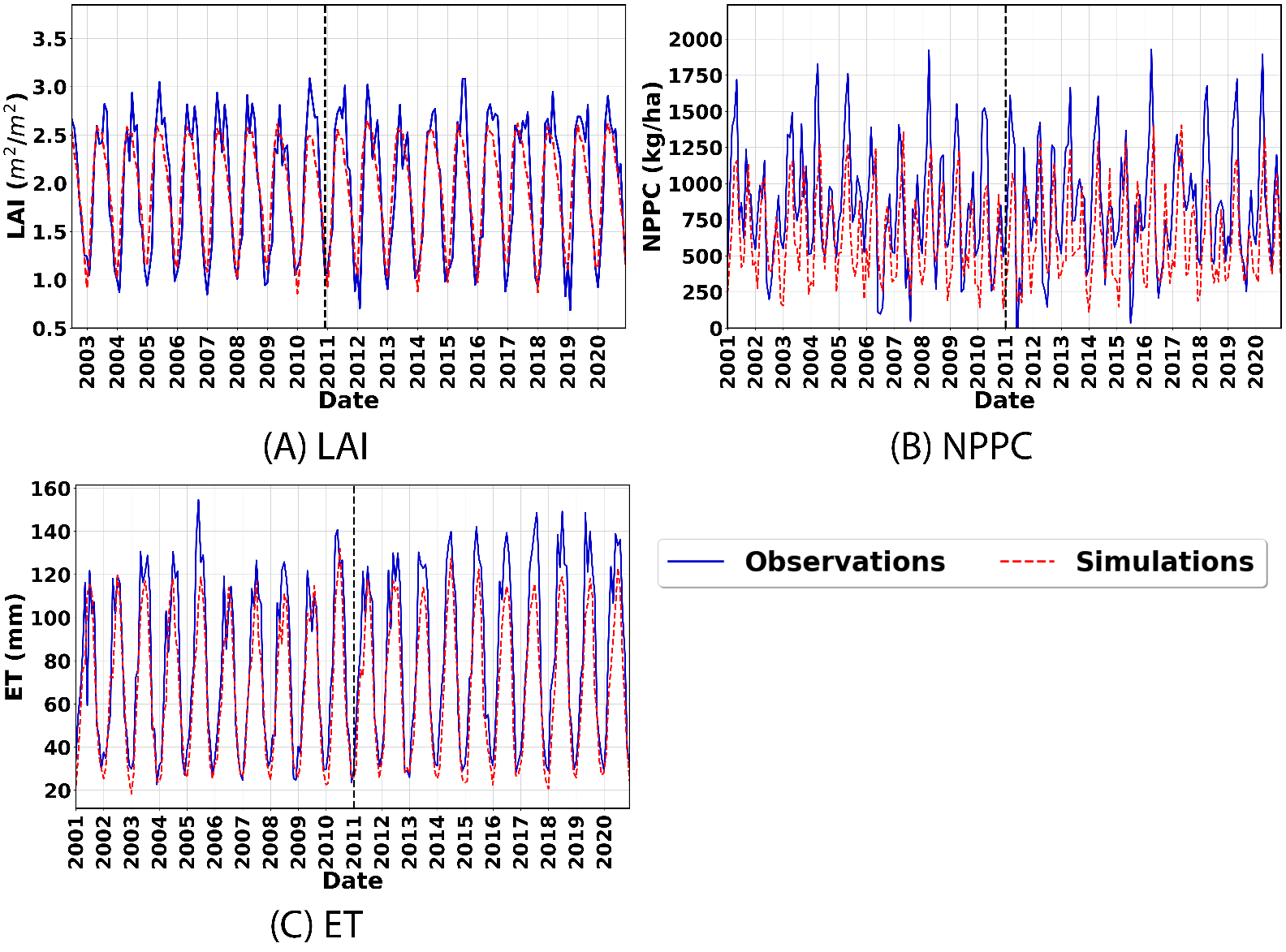


**Figure S3.** The assessment of the LAI (A), NPPC (B), and ET (C) simulations at the basin scale. The dashed lines mark the boundary separating the calibration and validation periods.


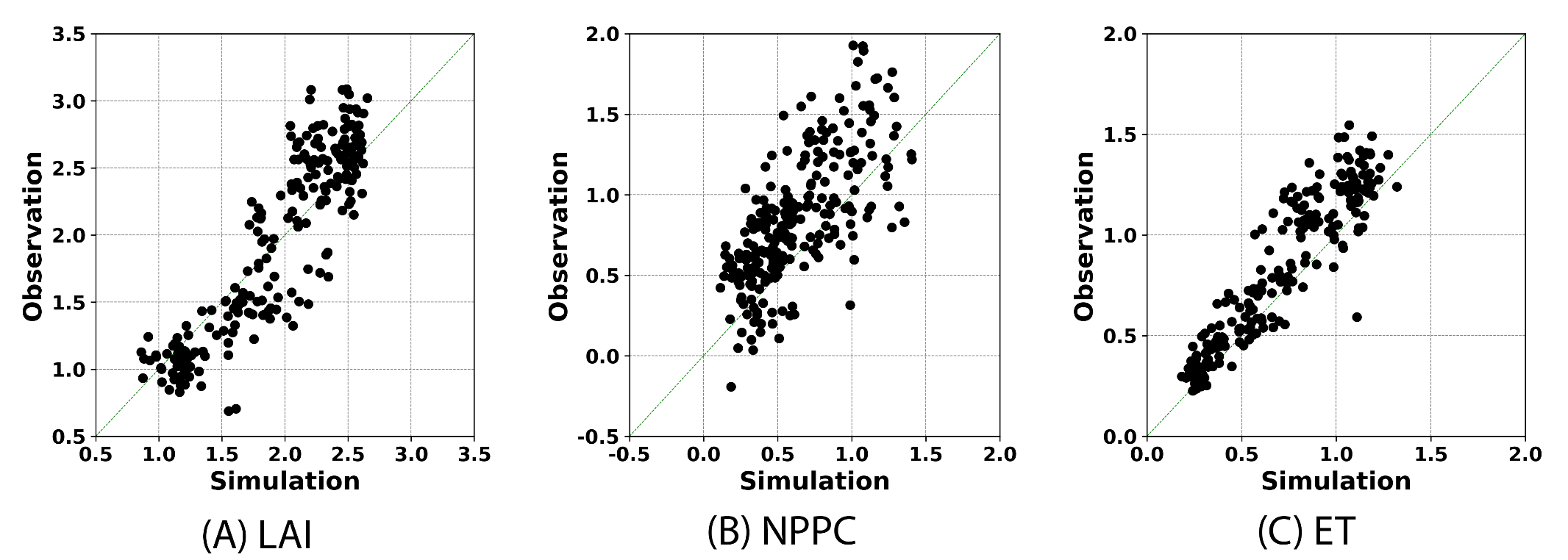


**Figure S4.** The scatter plots for assessing the LAI (A), NPPC (B), and ET (C) simulations at the basin scale.


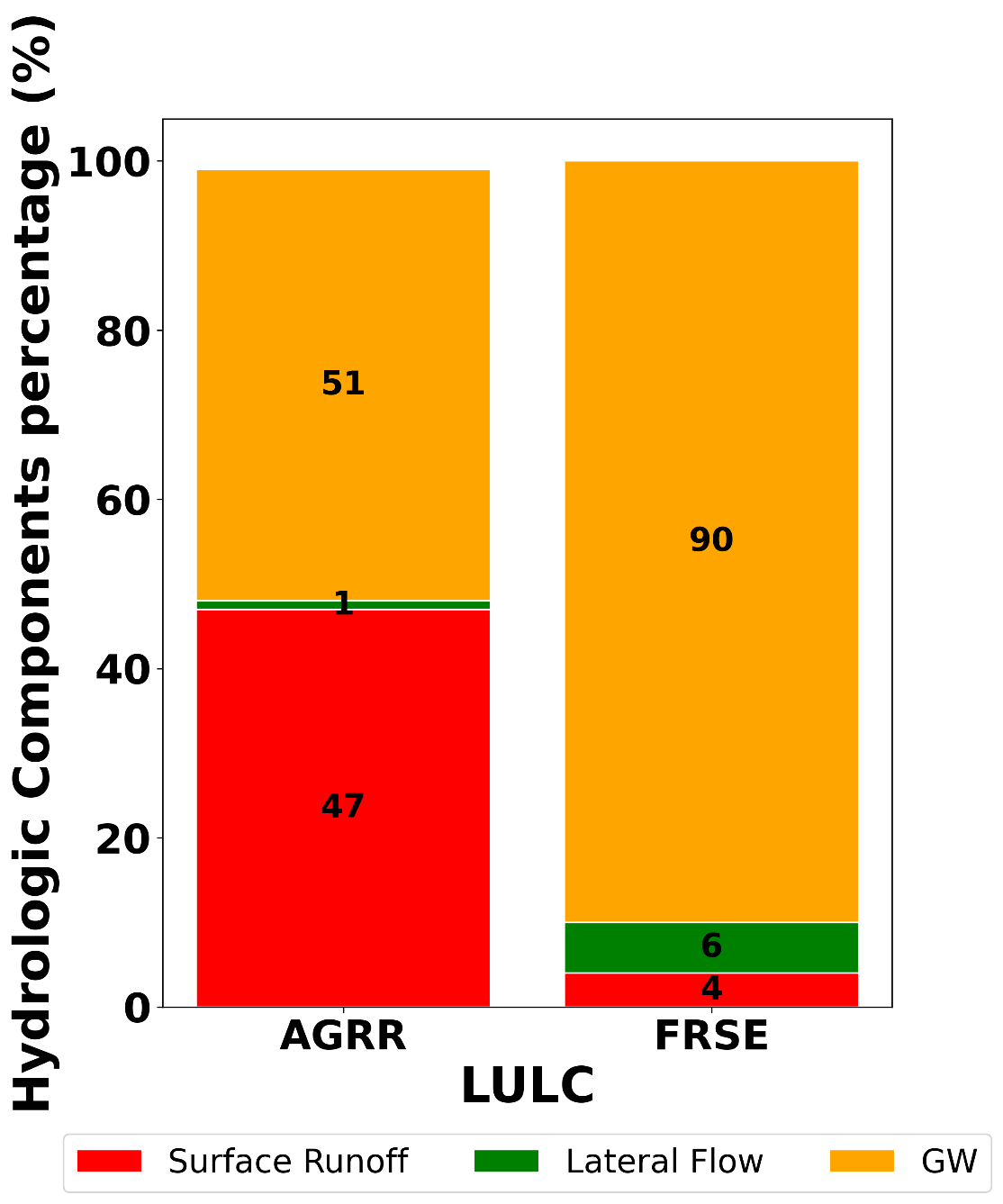


**Figure S5.** Assessment of dominant hydrologic pathways for all agricultural LULC (AGRR) and critical source areas for DOC transport within the evergreen forest (FRSE) LULC.


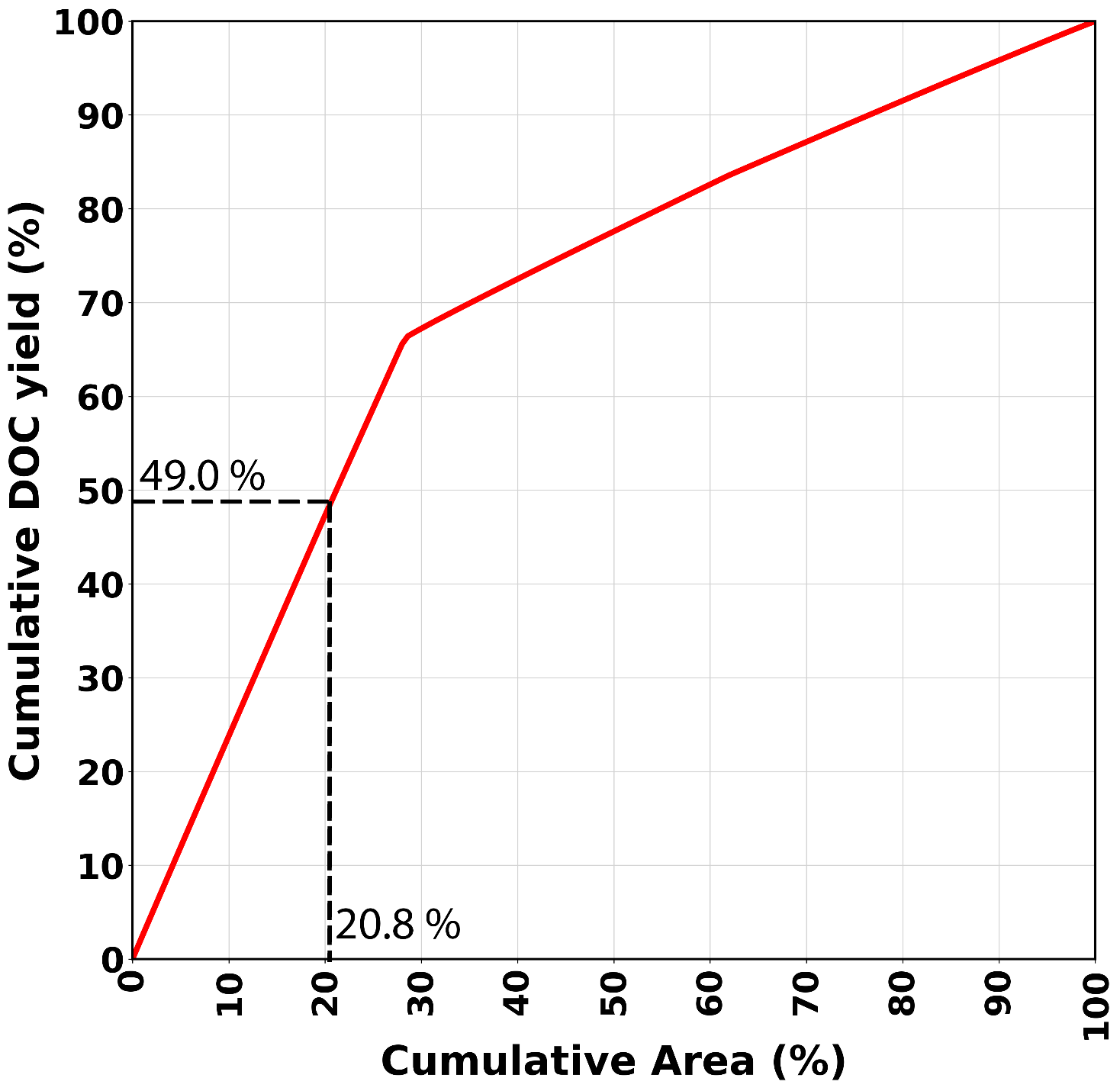


**Figure S6.** Distribution of DOC yields by evergreen forest area (%) in a Big Creek watershed. The y-axis dashed lines mark the threshold values (20% of the forest areas) and the x-axis dished lines mean the cumulative DOC yield percentage in the threshold area.
